# Monitoring of Dual CRISPR/Cas9-Mediated Steroidogenic Acute Regulatory Protein Gene Deletion and Cholesterol Accumulation Using High-Resolution Fluorescence *In Situ* Hybridization in a Single Cell

**DOI:** 10.3389/fendo.2017.00289

**Published:** 2017-10-25

**Authors:** Jinwoo Lee, Colin Jefcoate

**Affiliations:** ^1^Department of Cell and Regenerative Biology, University of Wisconsin, Madison, WI, United States; ^2^Endocrinology and Reproductive Physiology Program, University of Wisconsin, Madison, WI, United States; ^3^Molecular and Environmental Toxicology Center, University of Wisconsin, Madison, WI, United States; ^4^Molecular and Cellular Pharmacology, University of Wisconsin, Madison, WI, United States

**Keywords:** steroidogenic acute regulatory protein, CRISPR, fluorescence *in situ* hybridization, cholesterol, lipid droplets

## Abstract

Recent advances in fluorescence microscopy, coupled with CRISPR/Cas9 gene editing technology, provide opportunities for understanding gene regulation at the single-cell level. The application of direct imaging shown here provides an *in situ* side-by-side comparison of CRISPR/Cas9-edited cells and adjacent unedited cells. We apply this methodology to the steroidogenic acute regulatory protein (StAR) gene in Y-1 adrenal cells and MA-10 testis cells. StAR is a gatekeeper protein that controls the access of cholesterol from the cytoplasm to the inner mitochondria. The loss of this mitochondrial cholesterol transfer mediator rapidly increases lipid droplets in cells, as seen in StAR^−/−^ mice. Here, we describe a dual CRISPR/Cas9 strategy marked by GFP/mCherry expression that deletes StAR activity within 12 h. We used single-molecule fluorescence *in situ* hybridization (sm-FISH) imaging to directly monitor the time course of gene editing in single cells. We achieved StAR gene deletion at high efficiency dual gRNA targeting to the proximal promoter and exon 2. Seventy percent of transfected cells showed a slow DNA deletion as measured by PCR, and loss of Br-cAMP stimulated transcription. This DNA deletion was seen by sm-FISH in both loci of individual cells relative to non-target Cyp11a1 and StAR exon 7. sm-FISH also distinguishes two effects on stimulated StAR expression without this deletion. Br-cAMP stimulation of primary and spliced StAR RNA at the gene loci were removed within 4 h in this dual CRISPR/Cas9 strategy before any effect on cytoplasmic mRNA and protein occurred. StAR mRNA disappeared between 12 and 24 h in parallel with this deletion, while cholesterol ester droplets increased fourfold. These alternative changes match distinct StAR expression processes. This dual gRNA and sm-FISH approach to CRISPR/Cas9 editing facilitates rapid testing of editing strategies and immediate assessment of single-cell adaptation responses without the perturbation of clonal expansion procedures.

## Introduction

The capacity to resolve individual RNA species in single cells by single-molecule Fluorescence in Situ Hybridization (sm-FISH) (1, 2) now provides the means to examine the CRISPR/Cas9 gene editing in single cells. Here, we describe a dual CRISPR/Cas9 cleavage of steroidogenic acute regulatory protein (StAR), the prime regulator of cholesterol metabolism, in Y-1 adrenal cells and MA-10 testis cells. We used direct sm-FISH to compare StAR expression in dual-transfected CRISPR (+) cells to non-transfected (NT) adjacent cells. The goal was to separate the timing, respectively, of the transfection, editing, and gene expression processes. We also measured the subsequent adaptation resulting from the loss of StAR function. We demonstrated dramatic increases of lipid droplets (LDs) that mimic the human adrenal deficiency condition (3).

This single-cell detection depends on sm-FISH, which uses multiple fluorescent 20-base oligomers (4) to detect primary transcripts (p-RNA) and spliced transcripts (sp-RNA) at gene loci and, then, to detect mRNA as single molecules in the cytoplasm (1, 2). cAMP analogs extensively induce these StAR RNA species in the Y-1 adrenal and MA-10 testis cells that we used here (5, 6). The Y-1 cells are distinguished by basal StAR mRNA expression, which was sufficient for maximum stimulation by cAMP within 10 min of steroid synthesis (7). sm-FISH imaging of StAR expression showed that the loci responded asymmetrically to cAMP stimulation within asynchronous cell populations. Stimulation of StAR transcripts at the gene loci not only increased the levels of different types of RNA but also decreased inter-cell differences.

Understanding the effects of CRISPR/Cas9 on StAR expression requires an appreciation of the editing process. The CRISPR/Cas9 technology was developed from bacterial adaptive immune systems (8–10). Cas9 is an RNA-guided DNA endonuclease that fuses with a guide RNA (gRNA). The gRNA includes a four-base endonuclease cleavage sequence and a Cas9 recognition site [protospacer adjacent motif (PAM)] at the 3′end (11–13). The association of Cas9 and gRNA directs specific localization to complementary DNA sequences selected for gene editing (14, 15). Here, we used a dual Cas9 vector strategy in which mCherry and GFP expression marked the respective deliveries of the 5′- and 3′- gRNA sequences. The guided Cas9 creates a double-stranded break (DSB) 3 bp upstream of the PAM sites, within the gRNA hybridized sequence (13, 16, 17). The dual cleavages this design provided lead to an excision and re-ligation to produce an edited StAR gene lacking the early proximal promoter, exon 1, and intron 1. This deletion removed the possibility of functional mRNA expression. We directly assessed the deletion by measuring the deletion time course of deletion by PCR amplification of the targeted StAR gene segment and by probing the edited StAR DNA segment with sm-FISH after RNase removal of all RNA. We compared this targeted StAR deletion to a non-targeted region of the StAR locus (exon 7) or to another similarly expressed gene (Cyp11a1 loci). We used this Cas9 procedure to examine the immediate consequences of StAR deletion. The StAR transfer of cholesterol from LDs to the cleavage enzyme, Cyp11a1, located on the inner face of the inner membrane, is integrated with the cleavage of cholesterol esters (CEs) by hormone-sensitive lipase (HSL), under control of protein kinase A (PKA) (18). In StAR^−/−^ mice and human deficiency, the loss of steroidogenesis in the adrenal glands, testes, and ovaries is matched by large accumulations of CE (3, 19).

In this report, we show that editing was extensive within 12 h and that cholesterol trafficking was then rapidly redirected from intra-mitochondrial oxidation to the formation of cytoplasmic CE accumulations. The cholesterol captured as esters in these accumulations was much greater than the small amounts diverted from StAR-dependent steroid synthesis. StAR has been linked to several cell types to cholesterol export (20), to transcription controlled by hydroxy-metabolites through liver X receptors (LXR) (21, 22), and to other mitochondrial processes (23).

These direct sm-FISH analyses of editing can be completed within 24 h of transfection. The speed this procedure provides the opportunity for a relatively quick optimization of the CRISPR/Cas9 editing strategy, notably for gRNA selection and validation. We have designed gRNA sequences with an online CRISPR design tool and used the fast algorithm Cas-OFFinder, to searches for potential off-target sites of Cas9 (24). Various PCR-based methodologies are available for single-guide RNA (sgRNA) validation, including the following: mismatch cleavage assays (25), indel detection by amplicon analysis (IDAA) (26), and digital PCR (27). However, a complete workflow of gRNA validation in single-cell colony expansion is time intensive. This direct combined sm-FISH and CRISPR/Cas9 strategy provided an intermediate step in the isolation of appropriately targeted clonal lines.

## Materials and Methods

### Cell Lines

The MA-10 mouse Leydig tumor cell line (a gift of Dr. Mario Ascoli) was derived from the Leydig tumor, M5480P (28). The Y-1 cell line (a gift of Dr. Bernard Schimmer) was derived from a mouse adrenocortical tumor (29). General procedures for cell culture were as described previously (1). A day before the experiment, approximately 3–6 × 10^5^ cells were seeded onto the coverslip (Corning) coated with ploy-Lysine (Sigma) in wells of a six-well plate (Corning).

### Real-time RT-PCR and Genomic DNA-PCR for Analysis of StAR

Primer design and cDNA synthesis were performed as previously described (1, 30). qPCR was performed using BioRad CFX96™ Real-Time PCR Detection System. The qPCR protocolfor StAR expression time courses was done as follows: initial denaturation at 95°C for 3 min, followed by 40 cycles of 10 s at 95°C and 30 s at 60°C. Primer sequences are as follows: Star E1/I1, 5′-AGACATATGCGGAATATGAAAGGTG-3′ and 5′-CCCAAGAGCTTTCCCACAAA-3′; E5/E6, 5′-GAGTGGTGTCATCAGAGCTGAAC-3′ and 5′-TGAGTTTAGTCTTGGAGGGACTTCC-3′. Genomic DNA extraction was done with TRIzol following the manufacturer’s protocol. The genomic DNA PCR protocol was done as follows: initial denaturation at 95°C for 3 min, followed by 35 cycles of 15 s at 95°C, 30 s at 58°C, and 150 s at 72°C. Primer sequences are as follows: 5′-CCTCTGCACAATGACTGATGACT-3′ and 5′-GGATGGGTCAAGTTCGACGTCGG-3′. PCR products were separated on a 0.5% agarose gel.

### Design of sgRNA Sequence

pSpCas9 (BB)-2A-GFP (Addgene plasmid # 48138) and pU6-(BbsI)_CBh-Cas9-T2A-mCherry (Addgene plasmid # 64324) were used for the sgRNA expression plasmids (31). pSpCas9 (BB)-2A-GFP express a chimeric gRNA plus EGFP and human codon-optimized Cas9. EGFP was replaced into Cherry in pU6-(BbsI)_CBh-Cas9-T2A-mCherry. The components of type II CRISPR/Cas9 system crRNA and tracrRNA were fused to generate a sgRNA. For the *S. pyogenes* system, the target sequence leads a 5′-NGG PAM and the guide sequence pairs with the opposite strand at ~3 bp upstream of the PAM. The targeted sequences are as follows: StAR exon 2 GGTGGATGGGTCAAGTTCGACGT CGG and StAR promoter AGTCATCAGTCATTGTGCAG AGG. To clone the guide sequence into the sgRNA scaffold, we designed guide sequences with online CRISPR design tools.^1^^,^^2^ To search for potential off-target sites of Cas9, we used Cas-OFFinder.^3^

### Plasmid Preparation

Digested plasmids [plasmid (1 µg), FastDigest Bbsl (1 µl), FastAP (1 µl), 10× FastDigest Buffer (2 µl), ddH_2_O (total 20 µl), 30 min, 37°C] were purified using QIAquick Gel extraction kit. The oligo pairs encoding the guide sequences were annealed and ligated into the plasmids. We prepared the mixture [sgRNA left (100 µM, 1 μ), sgRNA right (100 µM, 1 µl), 10× T4 ligation buffer l (1 µl), T4 PNK (1 µl), ddH_2_O (total 10 µl)] for phosphorylating, and annealing the sgRNA oligos in a thermocycler (37°C for 30 min; 95°C for 5 min; ramp down to 25°C at 5°C min^−1^). We set up a ligation reaction (Bbsl), and the mixture were incubated for 10 min at RT. We also prepared a no-insert pSpCas9 (BB)-only negative control for ligation. DH5 alpha cells were used for transformation. The product (2 µl) was added to ice-cold DH5 alpha cells (50 µl), incubated the mixture on ice for 30 min, heat-shocked the cell mixture at 42°C for 45 s, and returned it immediately to ice for 2 min. Then, we added 500 µl of SOC medium and incubated for 1 h before plating the cells onto an LB plate containing 100 µg ml^−1^ ampicillin. After an overnight incubation at 37°C, we picked 10 colonies to check for the correct insertion of the sgRNA. We ran the colony PCR reaction with the guide sequence (forward) and a primer on the plasmid backbone (reverse: CAG_enhancer region).

### Transfection

DNA was transfected with 7.5 µl of Lipofectamine 3000 reagent, 5 µl P3000, and 2.5 µg of plasmid DNA cocktail following the manufacturer’s instructions. CRISPR (+) cells under conditions used here are present in 25–30% of the cells 24 h post-transfection. We analyzed all NT cells in randomly selected fields. The smaller proportion of CRISPR (+) cells necessitates analysis of all GFP (+) cells in wider fields.

### Single Molecule Fluorescence *In Situ* Hybridization (sm-FISH)

The term sm-FISH has been used to distinguish the use of a probe set consisting of 40 fluorescent 20mers. The RNA probe sets for StAR were generated by using the Stellaris probe designer.^4^ Samples were prepared according to a previously described method (1). Freshly prepared StAR p-RNA (Quasar-570, Biosearch Technology), StAR sp-RNA (Quasar-670, Biosearch Technology), StAR 3′ EU (Quasar-570, Biosearch Technology), and Cyp11a1 p-RNA (Quasar-670, Biosearch Technology) probe sets and antibody for StAR were used. A clean coverslip was placed over the sample to prevent drying of the RNA hybridization solution [10% dextran sulfate (Sigma), 10% deionized formamide (Ambion), 2× SSC] during the incubation. Samples were incubated in a dark humidified chamber at 37°C overnight. After a 30-min wash in wash buffer, samples were incubated in DAPI nuclear stain (wash buffer with 5 ng/ml DAPI) to counterstain the nuclei for 30 min. In the case of the combined FISH, an extra washing step is needed with the secondary antibody. Samples were processed according to as previously described (1).

The sm-FISH method has been shown to be effective at visualizing well-expressed mRNA transcripts in most cells. Glass coverslips with plated cells were washed with sterile PBS and placed in six-well plates. Cells were plated on glass coverslips, fixed with 0.4% paraformaldehyde, stored at 4°C in 70% ethanol overnight, and hybridization was performed the next day. For single mRNA detection, sm-FISH probe sets consist of multiple singly labeled oligonucleotides (generally 30–48 bases long) designed to hybridize along targeted RNA transcripts. Hybridization was performed in a dark humidified condition. Samples were re-suspended in 2× SSC and added GLOX buffer without enzymes for equilibration, incubated, and then re-suspended in GLOX buffer with enzymes (glucose oxidase and catalase). Again, antifade reagent was used prior to Z-stack imaging.

For DNA sm-FISH, cells were treated with RNase A at 37°C for 1 h, washed and equilibrated with PBS for 5 min before dehydration by consecutive 5 min incubations in 70, 85, and 100% ethanol. After air-drying, cells were heated at 80°C for 5 min in hybridization mixture (50% formamide, 10% dextran, 0.5 µM EDTA, and 4× SSC), and then washed using an ethanol series (ice old 70, 80, and 95%). After air-drying, cells were placed in RNA hybridyzation with probes for overnight.

### Oil Red O (ORO) (Lipid Stain) Staining

Oil Red O stock solution was prepared by dissolving 0.3 g of ORO (MP Biomedicals, OH, USA) in 100 ml of isopropanol. ORO working solution was prepared by mixing three parts (30 ml) of ORO stock solution with two parts (20 ml) of water and filtered through 0.2 µm filters. Cells were washed with water and incubated with 60% isopropanol for 5 min. After 5 min, the wells were completely dry. ORO working solution was added to the cells, and the cells were incubated for 10 min in the solution. Stained cells were washed three times with water. Again, the antifade reagent was used prior to Z-stack imaging. ORO is a fat-soluble dye and can be used for fluorescence microscopy wherein a lipid environment has enough fluorescence (32).

### Image Acquisition and Analysis

To detect and visualize p-RNA, sp-RNA, and mRNA, we used the Olympus wide-field fluorescence microscope (Model IX81) and the Nikon’s Structured Illumination Microscope (N-SIM) for higher resolution images according to a previously described method (1, 33). We used two microscope settings representing high and low sensitivity to compare p-RNA and sp-RNA at loci under, respectively, basal conditions and after 1 and 3 h of stimulation by Br-cAMP (Figure S1B in Supplementary Material). The high sensitivity setting is also used to image mRNA, which appears as single molecules. The N-SIM uses different optical processing methods but provided results that were fully consistent with the Olympus IX81. Five fluorescence imaging filter set (DAPI, GFP, Orange, mCherry, and Red) was used for IX81 in this study. GFP (488ext/507emi nm) and mCherry (587/610 nm) channels were used for the detection of Cas9-GFP and Cas9-mCherry. Orange (538/559 nm, pseudocolored green) and red (618/637 nm, pseudocolored red) channels were used for the detection of StAR p-RNA and sp-RNA/mRNA. Yellow color indicates colocalization of p-RNA and sp-RNA. We used the “StAR Search” software developed by the Arjun Raj lab at University of Pennsylvania.^5^ Statistical significance was determined by Student’s *t*-test or ANOVA; *p* < 0.05 was considered statistically significant where *p < 0.05, **p < 0.01, and ***p < 0.001. Data were analyzed by using the PRISM software (San Diego, CA, USA).

## Results

### Experimental Design for CRISPR/Cas9 Deletion from the StAR Gene

**Figure 1:**
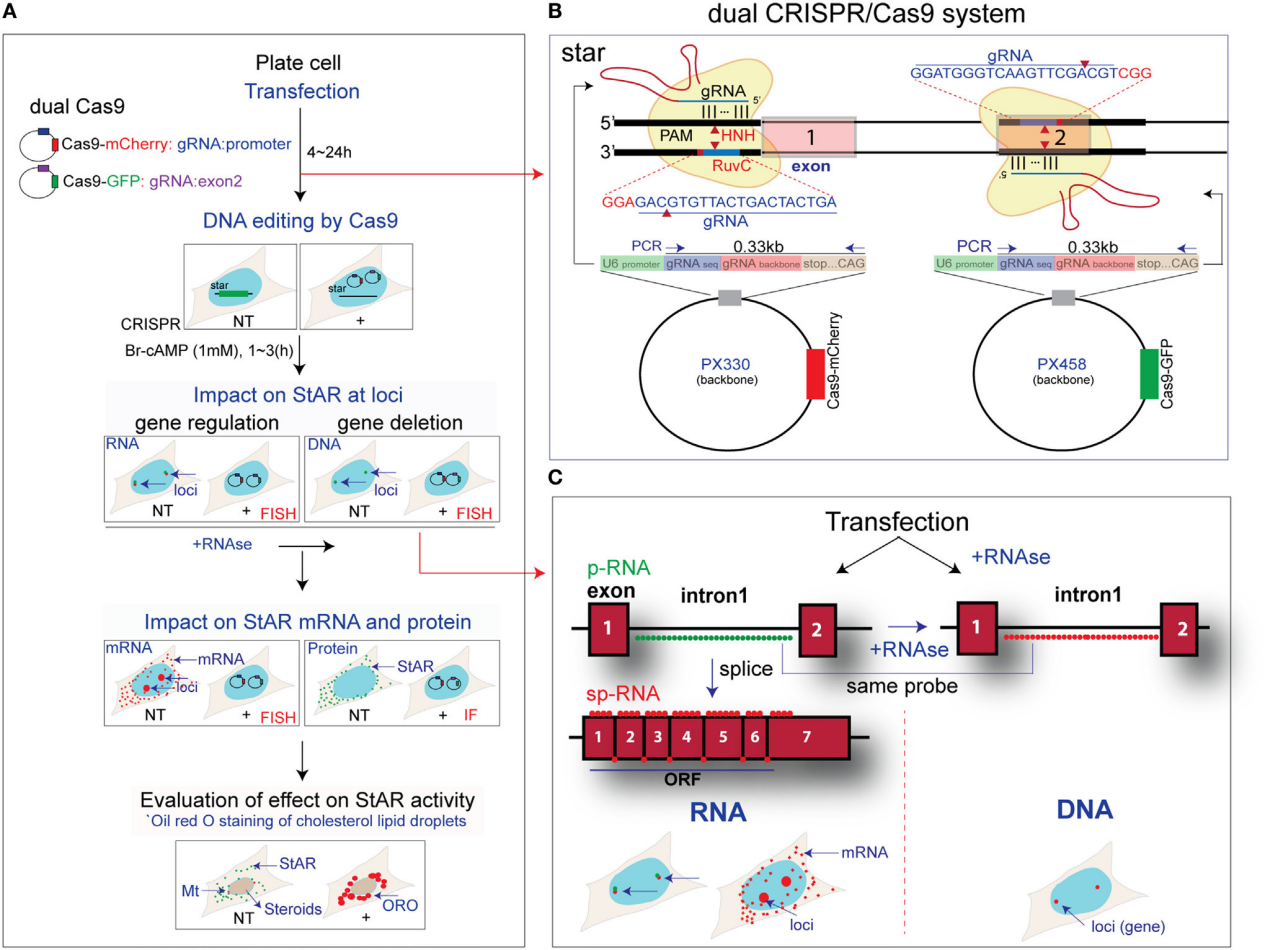
Design for CRISPR/Cas9 editing of steroidogenic acute regulatory protein (StAR). **(A)** The sequence of steps used for dual CRISPR/Cas9 deletion of the StAR DNA from Y-1 and MA-10 cells. Transfection and DNA editing by Cas9 were assessed by impacts on StAR RNA at loci, on StAR mRNA and protein in the cytoplasm, plus evaluation of effects on StAR activity based on Oil Red O (ORO) staining of cholesterol in lipid droplets (LDs). Side-by-side comparison of NT and CRISPR (+) cells was performed. **(B)** Dual CRISPR/Cas9 design for StAR. Two gRNAs were used to generate a large deletion of the gene. CRISPR (+) cells expressed both gRNAs and Cas9 conjugated with mCherry (PX330) or GFP (PX458), which indicates gRNA expression. **(C)** Characterization of editing: high-resolution fluorescence *in situ* hybridization probes were used for identification of altered StAR p-RNA and sp-RNA at loci during CRISPR/Cas9 editing. Targeted StAR DNA at gene locus was also visualized by the p-RNA probe for intron 1 after the RNase treatment. Single molecules of mRNA were detected by sp-RNA probes. StAR antibody was also added for immunofluorescence (IF) images.

This study has two general goals; first, to separate the timing of CRISPR/Cas9 editing of StAR through the temporal resolution in individual cells of transfection, editing, and gene expression; second, to measure the direct adaptation of the cells due to loss of StAR function (Figure 1A). The progress in editing was measured in whole cultures by PCR analysis of the targeted segment of StAR DNA and then in single cells by direct sm-FISH imaging. We examined both the StAR DNA sequence that was targeted by CRISPR/Cas9 and the associated impact on StAR expression. We tested both basal, and Br-cAMP-stimulation of StAR expressed either as RNA at gene loci or as mRNA and protein in the cytoplasm. The final adaptation phase was indicated by fluorescence imaging of significant increases in CEs that form the major constituents in cytoplasmic LDs and also accumulate in late endosomes (34). These accumulations were readily detected in single cells by ORO, which delivered a low-intensity fluorescence (32). ORO (+) droplets were increased extensively in CRISPR (+) cells. We measured the time course of this response in individual cells in relation to the StAR RNA editing responses.

CRISPR/Cas9 editing produces sequence deletions that remove expression or activity when two gRNA sequences target double-strand DNA cleavage sites that span a functionally essential part of the gene locus. The StAR gene sequence between these two gRNAs is lost, and ligation between the 5′- and 3′-ends restores the integrity of the gene. We have selected a 5′ target that overlaps C/EBPβ-binding site (−90 ~ −80 bp) (35) in the StAR promoter and a site on the reverse strand at the end of the exon 2 region. A deletion of key promoter elements then ensues, including the transcription StAR site, exon 1, intron 1, and most of intron 2 (Figure 1B). To test the efficiency of gRNA and to visualize the impact of gene deletion, we have combined this dual CRISPR/Cas9 strategy with sm-FISH analyses of the various steps in gene expression.

We have previously reported a set of sm-FISH oligomers (1, 2) that image specific segments of the StAR gene (intron 1 and the 3′end of exon 7) and equivalent sequences in RNA transcription products. We have resolved p-RNA and sp-RNA of StAR gene loci and single mRNA molecules in the cytoplasm (Figure 1C). Although mRNA expression in individual cells was variable, image analyses of sets of adjacent cells provided a mean expression as copy numbers per cell that matched equivalent qPCR analyses based on several million cells in a culture well (1, 2). Here, we have applied these sm-FISH probes to *in situ* single-cell analysis of CRISPR/Cas9 editing.

The StAR sm-FISH images obtained from Y-1 adrenal and MA-10 testis cells differ by over 50-fold ranging. We have previously reported single hybridizations for StAR gene DNA, for each resolved mRNA molecule and minimum p-RNA expression in basal Y-1 loci (2, 33). Peak unresolved fluorescence for, respectively, p-RNA and sp-RNA is seen in these loci after 1 h stimulation by Br-cAMP. StAR protein expression determined by immunohistochemistry has a similar range of expression, which corresponds to accumulation in mitochondria. The comparison of image intensities required the adjustment of microscope settings (Figures S1A–C in Supplementary Material). We also showed that the 3D distribution of loci and mRNA in these cells was appreciably different. Fortunately, the active StAR loci were found close to the nuclear midline thus facilitating detection (*Z* axis). Most of the mRNAs were found closer to the adherent surface in the widest part of the cell (Figure S1D in Supplementary Material). Optimum sensitivity for single DNA/RNA molecules was obtained with single probe sets where pixels can be directly counted with the aid of an imaging program (star search) (Figure S1E in Supplementary Material).

### Characterization of StAR Expression in Untransfected Y-1 Cells

**Figure 2:**
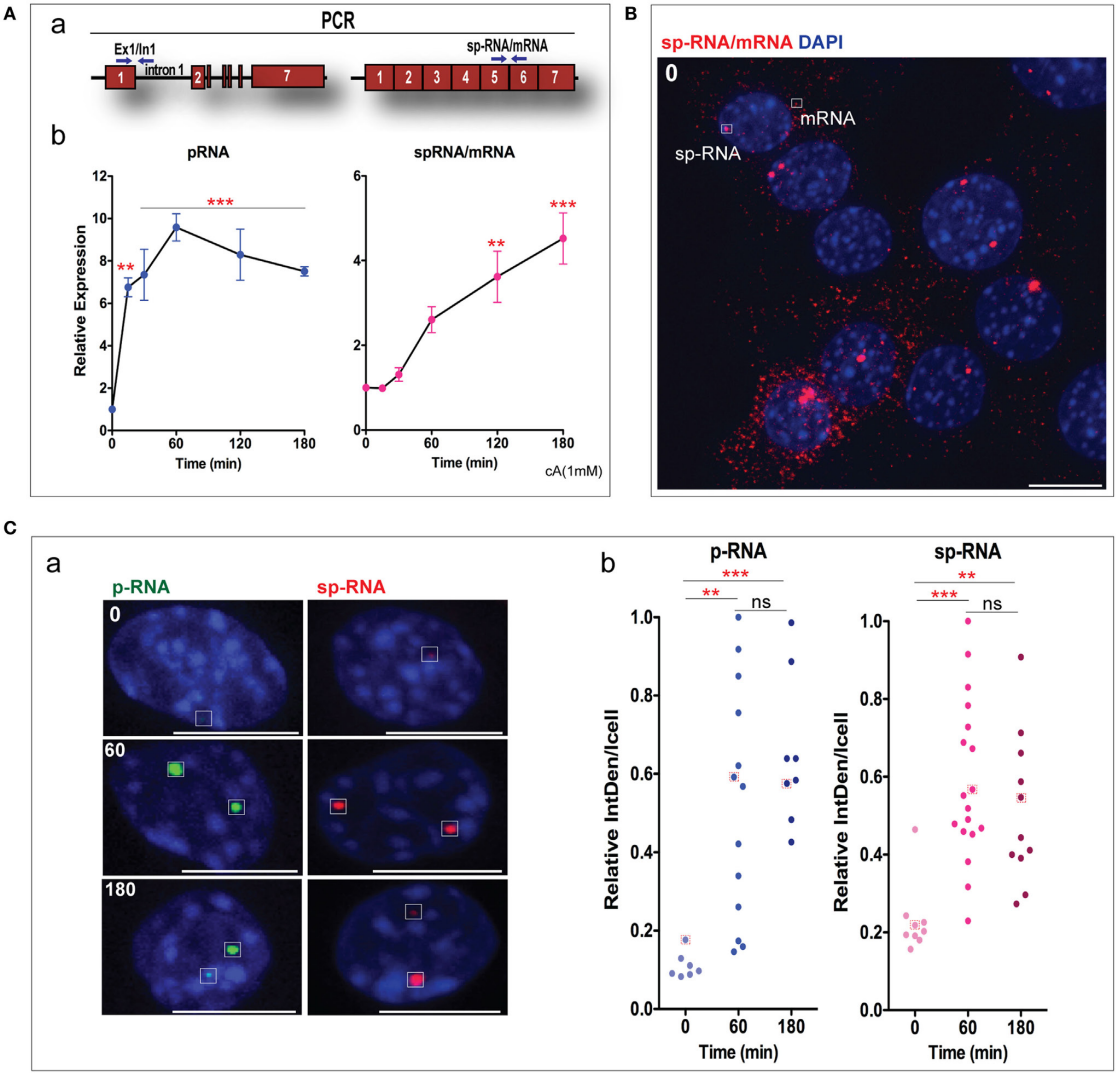

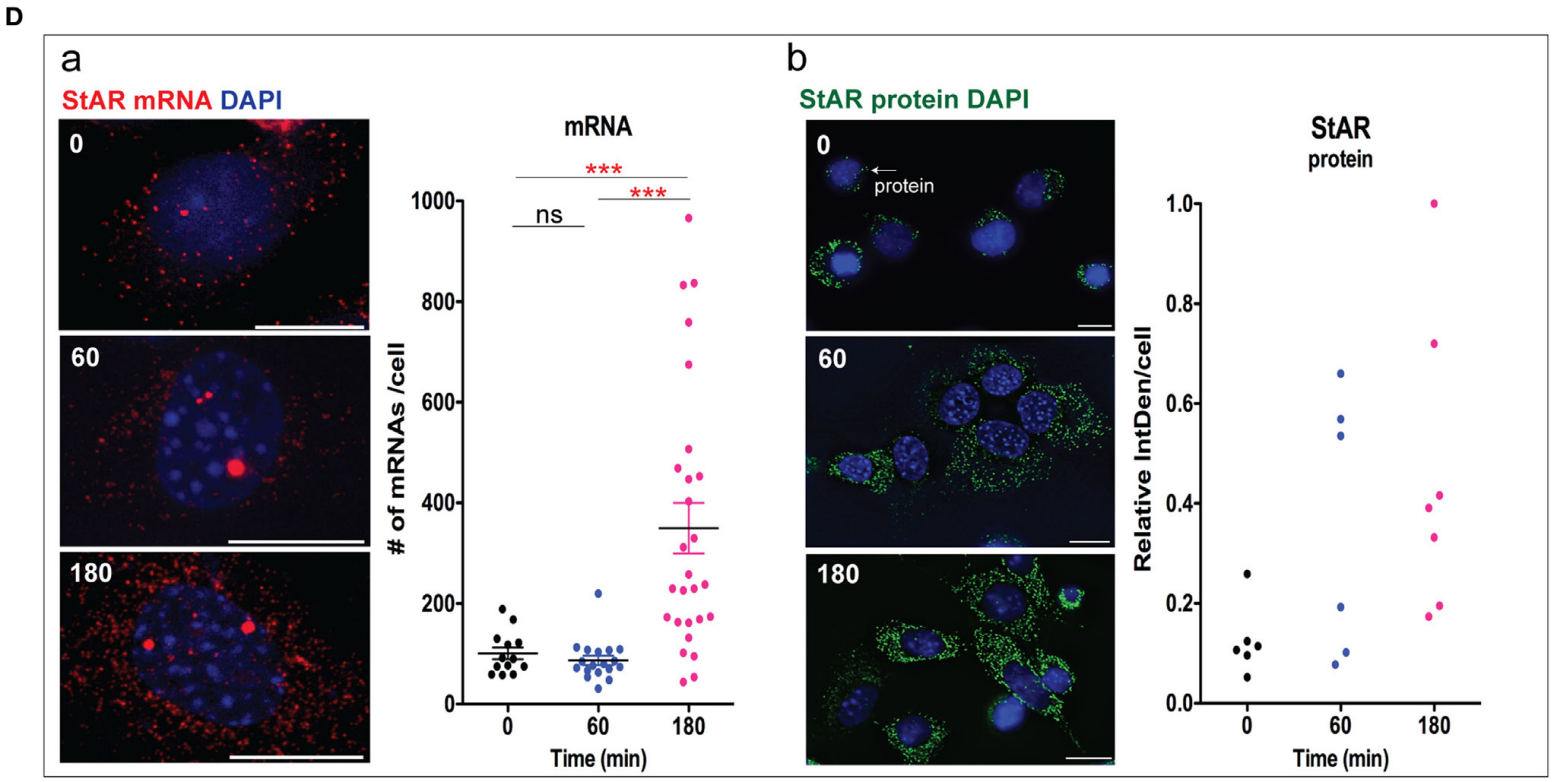
Characterization of steroidogenic acute regulatory protein (StAR) expression in Y-1 cells. **(A)** qPCR analysis of StAR transcription in Y-1 adrenal cells after stimulation by Br-cAMP. (a) Location of PCR primers to detect the primary transcripts [pRNA(E1/I1)] and spliced transcripts [spRNA(E5/E6)]. (b) Time course for the stimulation of StAR RNA as determined by q-PCR. **(B)** High sensitivity sm-FISH detection of basal expression of sp-RNA at loci and mRNA in cytoplasm. **(C)** (a) Low sensitivity paired sm-FISH images of the time-dependent appearance of p-RNA and sp-RNA at loci after stimulation by Br-cAMP (1 mM). (b) Relative integral density (IntDen) was measured based on the integrated density subtracted with background readings. **(D)** High sensitivity sm-FISH of StAR mRNAs (a) and IF of protein (b) after 1 and 3 h Br-cAMP (1 mM) stimulation. The “Star search” program was used for counting fluorescence of mRNA and protein immunohistochemistry in individual cells. Error bars show SEM. **p < 0.01, ***p < 0.001; ns, not significant by ANOVA with *post hoc* Tukey. Scale bar represents 10 µm.

To assess the impact of CRISPR/Cas9 editing in Y-1 cells, we first used q-PCR to characterize the normal expression of StAR p-RNA and sp-RNA after Br-cAMP stimulation. p-RNA increased 10-fold over 1 h to a steady state, while sp-RNA showed a delay of about 20 min [Figure 2A (a,b)]. We previously characterized the delay in sp-RNA as a pause in elongation at the end of the terminal exon that is coupled with splicing (1, 33). This increase is biphasic, corresponding first to the generation of sp-RNA from the splicing of p-RNA at the gene locus, and then to a slower process that corresponds to export of the processed mRNA from the loci to the cytoplasm. The primary p-RNA StAR transcripts were only found in the nucleus at the gene loci (1, 2).

Before any stimulation, Y-1 cells showed a consistent basal expression of p-RNA and sp-RNA/mRNA, which were unresolved when measured by q-PCR. Sm-FISH resolved this expression to highly variable levels of p-RNA and sp-RNA at the loci in the individual cells (Figure 2B). About one-third of the cells showed mRNA in the cytoplasm, and about one-third show none. There is little relationship between expression of sp-RNA at the loci and the mRNA in the cytoplasm. However, the expression at the loci turns over much more quickly than the cytoplasmic mRNA. In MA-10 cells, this basal level p-RNA and sp-RNA was undetectable (2, 36).

p-RNA and sp-RNA were expressed at the same loci in representative Y-1 cells at low sensitivity. Such robust expression was only visible in a few select basal cells. Two loci exhibited this robust expression in most Y-1 cells after of stimulation. There was no further increase at 3 h [Figure 2C (a)]. The dot blots indicated the range of expression at Y-1 loci under basal conditions, and with stimulations for 1 and 3 h [Figure 2C (b)].

We compared sm-FISH images of cytoplasmic mRNA under basal conditions and after 1 and 3 h of stimulation by Br-cAMP [Figure 2D (a)]. There was no net increase in cytoplasmic mRNA in the initial 1 h, despite substantial increases of sp-RNA in the loci. The cytoplasmic mRNA rose extensively at 3 h. We have previously shown (1, 33) that cytoplasmic mRNA increased to this steady-state level after 2 h. The sm-FISH image showed a significant increase in StAR protein at 1 and 3 h, which evidently preceded the increase in mRNA. This anomaly is even clearer in MA-10 cells where cytoplasmic mRNA is scarcely detectable at 1 h even as StAR protein exhibits peak translation. A major factor in this discrepancy is that StAR protein accumulates in mitochondria with slow turnover. By contrast, low levels of StAR mRNA, which can sustain peak StAR mitochondrial activity (7), may turnover more rapidly than the excess that induced by high concentrations of Br-cAMP.

### CRISPR Cas9 Editing of Basal and Stimulated StAR

We applied this sm-FISH analysis to basal and induced Y-1 cells 24 h after application of the dual CRISPR/Cas9 transfection. We examined NT and CRISPR (+) cells under basal conditions or after 1 h stimulation by Br-cAMP. This stimulation provides optimal responses of p-RNA and sp-RNA at the loci. mCherry and GFP fluorescence mark the expression of 5′ gRNA and 3′ gRNA, respectively. Their dual fluorescence is therefore indicative of CRISPR (+) cells. We first examined p-RNA and sp-RNA in Y-1 cells under basal conditions.

**Figure 3:**
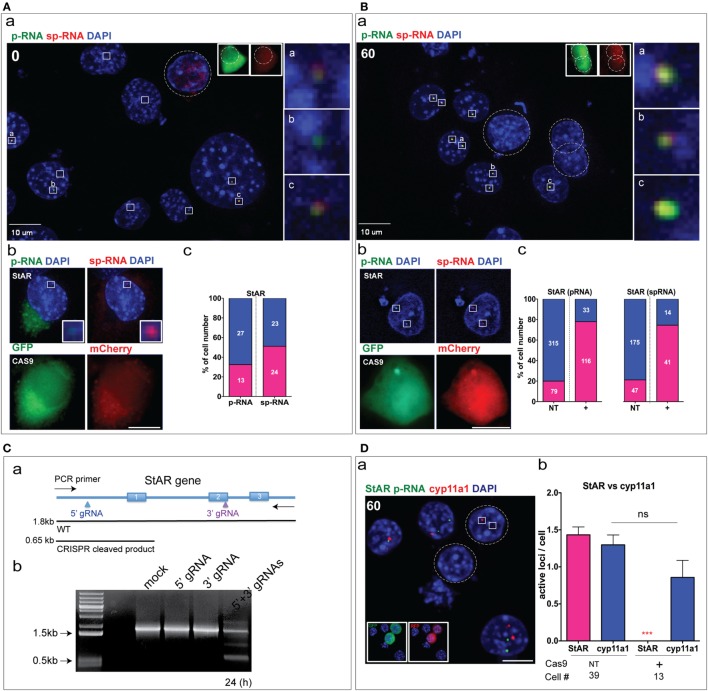
CRISPR Cas9 editing of basal and stimulated steroidogenic acute regulatory protein (StAR). **(A)** (a) CRISPR/Cas9 StAR deletion under the basal condition at the locus. The boxes indicate the active StAR loci (p-RNA, sp-RNA), and the dashed line (circle) shows the CRISPR/Cas9-positive cell. GFP and mCherry were used as a marker for gRNA expression. (b) a CRISPR (+) cell that retained an active StAR loci. (c) Percent of NT basal cells expressing p-RNA and sp-RNA at loci (the segment is shown in blue). Only 1/10 CRISPR (+) cells had detectable RNA at the locus. **(B)** (a) CRISPR/Cas9 StAR deletion was measured at the loci after 1 h stimulation with Br-cAMP (1 mM). (b) An example of a CRISPR (+) cell that retained active StAR loci. (c) Percent of non-transfected cells (NT, left) and CRISPR (+) cells (right) that expressed active StAR loci (p-RNA and sp-RNA) (segment shown in blue). **(C)** (a) PCR strategy to validate StAR gene deletion. (b) PCR amplification in wild type (WT) and transfected (24 h) cells with various CRISPR/Cas9 plasmids. **(D)** (a) Detection of p-RNA (StAR, cyp11a1) after 1 h stimulation with Br-cAMP in Y-1 cells. (b) Quantitation of active loci per cell for StAR and Cyp11a1 in NT cells and CRISPR (+) cells after 1 h Br-cAMP stimulation. Error bars show SEM. **p < 0.01, ***p < 0.001; ns, not significant by ANOVA with *post hoc* Tukey. Scale bar represents 10 µm.

High-sensitivity microscope settings were needed to detect the expression at these minimally active loci (Figure S2A in Supplementary Material). The transfected cells, which were identified by the expression of both mCherry and GFP, did not show expression at the level reached by the subset of higher-expressing basal cells (3/7 Figure 3A; inserts a–c). Only one CRISPR (+) cell, out of 10 examined cells, retained active StAR loci [Figure 3A (b)]. Overall, in the basal NT cells, 27/40 expressed p-RNA, and 23/47 expressed sp-RNA with most expressing both in varying ratios [Figure 3A (c)]. To confirm the specificity of these low-level CRISPR responses as well as the sensitivity of p-RNA and sp-RNA detection in the presence of a background, we examined expression in Y-1 cells with CRISPR/Cas9 transfection carried out in the absence of gRNAs (Figure S2B in Supplementary Material). Without gRNAs, the basal expression of p-RNA and sp-RNA was not hindered in the mCherry-positive cells. Nevertheless, the variability and low detection mean that still larger numbers of CRISPR (+) need to be processed to provide a statistically valid assessment of the CRISPR impact on basal Y-1 expression.

To improve the detection of StAR transcripts, we took advantage of the rapid stimulation of StAR p-RNA and sp-RNA at loci in Y-1 cells. After 1 h of Br-cAMP stimulation, p-RNA and sp-RNA were visible even at low sensitivity settings. In five representative NT cells, all loci expressed both p-RNA and sp-RNA with 3/5 exhibiting two active loci and 2/5 with a single active locus. No CRISPR (+) cell showed an active locus [Figure 3B (a)]. However, a lower proportion of CRISPR (+) cells retained active loci as shown [Figure 3B (b)]. Examination of all CRISPR (+) cells from several microscope fields showed that 33/149 (22%) retained some p-RNA expression, compared to 315/394 (80%) in NT cells [Figure 3B (c)]. Similar proportions were shown for sp-RNA [CRISPR (+) 14/55 (25%); NT 175/222 (78%)]. Thus, the ratio of primary to spliced StAR transcripts retained in the active CRISPR (+) loci appears similar to the NT loci, consistent with no effect of dual CRISPR/Cas9 on StAR splicing.

To establish dual sgRNA-directed gene deletion, we designed PCR primer sets that amplifies the StAR genomic DNA which we expect to be deleted by the dual CRISPR editing [Figure 3C (a)]. The full StAR gene product (WT, 1.8 kb) and deleted gene product [CRISPR (+), 0.65 kb] were separated on a 0.5% agarose gel. In the experiment, the deletion shown by a 0.65 kb band was about 25% at 12 h and at least 50% at 24 h [Figure 3C (b)].

To test the specificity of genome editing introduced by CRISPR/Cas9, we examined whether CRISPR (+) and NT cells maintained normal expression of Cyp11a1 p-RNA. StAR p-RNA was not suppressed in CRISPR (+) cells [Figure 3D (a)]. The four NT cells expressed Cyp11a1 in 3/4 cells, with two showing both loci and one only a single locus. Two CRISPR (+) cells showed one cell with two active Cyp11a1 loci and one without Cyp11a1 expression. Neither CRISPR (+) cell showed StAR expression. Overall, in 39 NT cells, StAR was expressed in an average of 1.4 loci per cell, with Cyp11a1 in 1.3 loci per cell. In CRISPR (+) cells, the Cyp11a1 expression decreased to 0.85 loci per cell, whereas StAR loci were undetected in these 13 CRISPR (+) cells [Figure 3D (b)].

### Application of gRNA to Manipulation of StAR Editing

**Figure 4:**
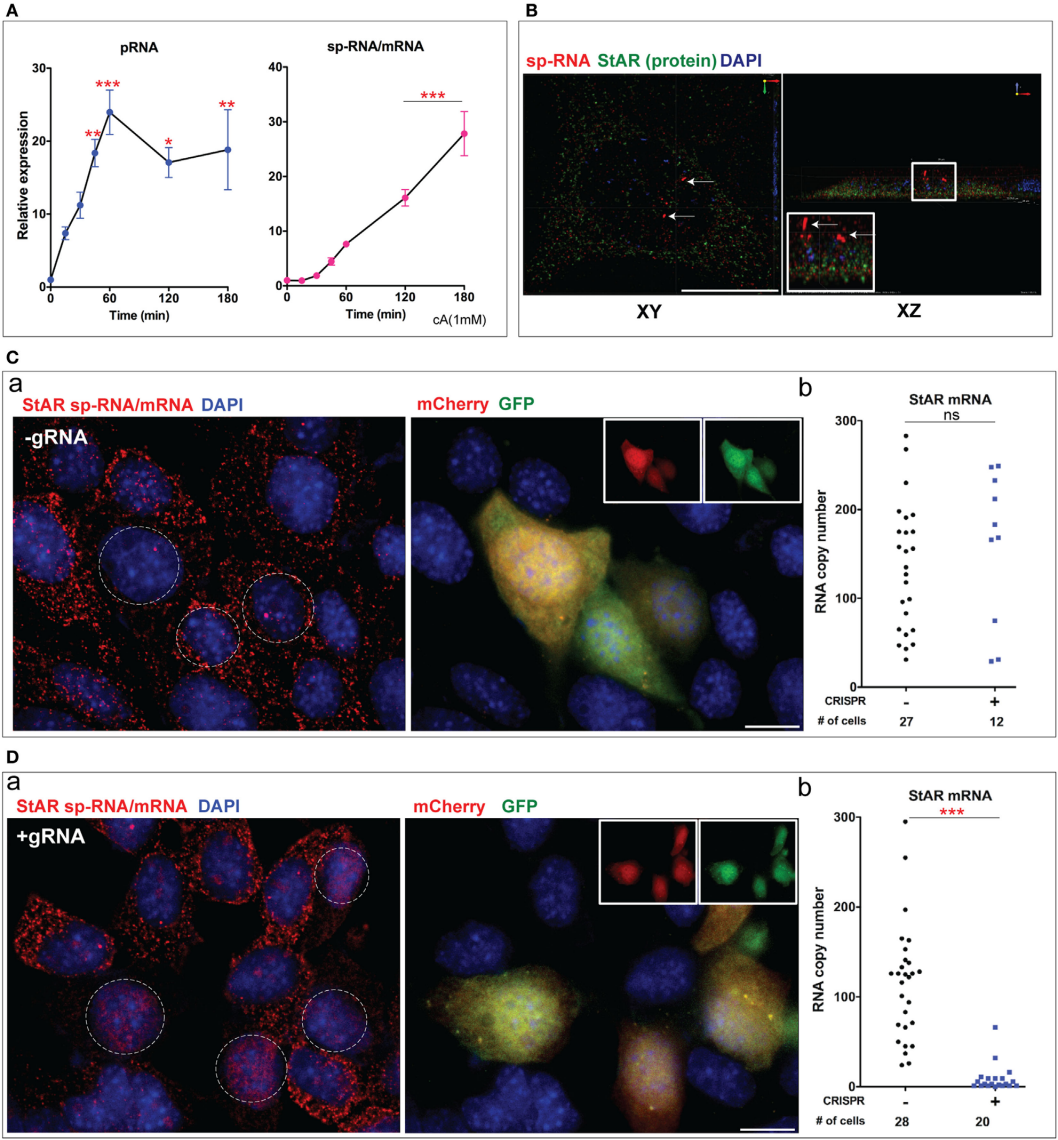
Role of gRNA in steroidogenic acute regulatory protein (StAR) editing; Br-cAMP stimulation of MA-10 Cells and suppression of mRNA. **(A)** qPCR analysis of StAR transcription in MA-10 testis cells. **(B)** XY and XZ projection of StAR mRNA and protein in MA-10 cells after 3 h of stimulation by Br-cAMP (1 mM). **(C)** CRISPR/Cas9 system without gRNA. (a) The high sensitivity of FISH image to StAR mRNA with sp-RNA probes. The dotted circles indicate the dual-transfected cells (left). GFP and mCherry’s dual fluorescence (right). (b) Quantitation of mRNA copy numbers in NT and GFP/mCherry (+) cells after 3 h of stimulation by Br-cAMP (1 mM). Expression differences were not significant in the *t*-test (ns). **(D)**. Dual CRISPR/Cas9 system with gRNAs. (a) High sensitivity to sp-RNA detection of mRNA (left) with GFP and mCherry detection of the transfected cells (right). Expression difference was significant by Student’s *t*-test ***p < 0.001. Scale bar represents 10 µm.

Single mRNA particles are harder to image than the accumulated RNA at loci, particularly in Y-1 cells, which slowly round up when stimulated by Br-cAMP. This cell rounding which is larger in Y-1 cells than in MA-10 cells arises from activation of PTPases that affect adhesion complexes (37). Overall, the stimulation of StAR p-RNA and sp-RNA in MA-10 cells by Br-cAMP was similar to that seen in Y-1 cells (Figure 2A) except that MA-10 cells were devoid of basal expression measured either by qPCR (Figure 4A) or sm-FISH (Figure S3 in Supplementary Material). Again, q-PCR showed that Br-cAMP stimulation was characterized by a steady state for p-RNA from 1 to 3 h. The formation of sp-RNA, which included increases first at the locus and then as mRNA in the cytoplasm, showed a 25-min delay.

The 3D spatial distribution of StAR loci, mRNA, and protein which is central to the sm-FISH assessment of gene expression was best seen in MA-10 cells. Thus, StAR protein and mRNA after 3 h of stimulation showed very characteristic 3D organizations (38) (Figure 4B). The StAR protein in these mitochondria is located in the matrix and is inactive in promoting cholesterol transfer to Cyp11a1. StAR is only active immediately after the translation of mRNA at the mitochondrial surface (7).

We next assessed whether this dual CRISPR/Cas9 system is needed for this removal of StAR sp-RNA/mRNA. MA-10 cells were transfected with the dual gRNAs, with each gRNA singly or without gRNAs and then assessed for mRNA expression. Without the gRNA, the three Cas9/GFP (+) cells retained the same StAR mRNA distribution as surrounding NT cells [Figure 4C (a)], with no statistical difference between NT and GFP (+) cells in the extent of expression [Figure 4C (b)]. Consistent with the Y-1 experiments, StAR mRNA was completely removed in the dual gRNA CRISPR (+) MA-10 cells [Figure 4D (a,b)].

Single 5′-gRNA/Cas9 or 3′-gRNA/Cas9 transfections of MA-10 cells for 24 h were marked by, respectively, mCherry or GFP. 5′gRNA CRISPR transfections produced similar effects after 24 h to the dual protocol. PCR analyses showed that neither single CRISPR/Cas9 produced any of the DNA deletion seen for the dual editing [Figure 3C (c)]. Out of eight mCherry (+) cells, six cells showed complete loss of transcription while two retained active StAR loci [Figure S4A (a,b) in Supplementary Material]. One of these cells with active loci also showed loss of mRNA, while one retained the mRNA. By contrast, the pattern of responses for 3′gRNA indicated much lower locus disruption [Figure S4B (a,b) in Supplementary Material]. Ten GFP (+) single transfections showed six that retained locus transcription, five of which expressed mRNA. Thus, half of the 3′gRNA-Cas9 transfections remain similar to the surrounding NT cells. Clearly, DSB introduced around the 5′ PAM site, located in the proximal promoter, more readily introduce mutations that remove StAR expression than DSB introduced around the 3′PAM site located in exon 2. The only difference between the 5′gRNA CRISPR (+) cells and the dual edit is that 1/8 of these cells retain both locus transcription and the full processing to mRNA.

### Detection of DNA Deletion from the StAR Loci of Y-1 Cells by Cas9

**Figure 5:**
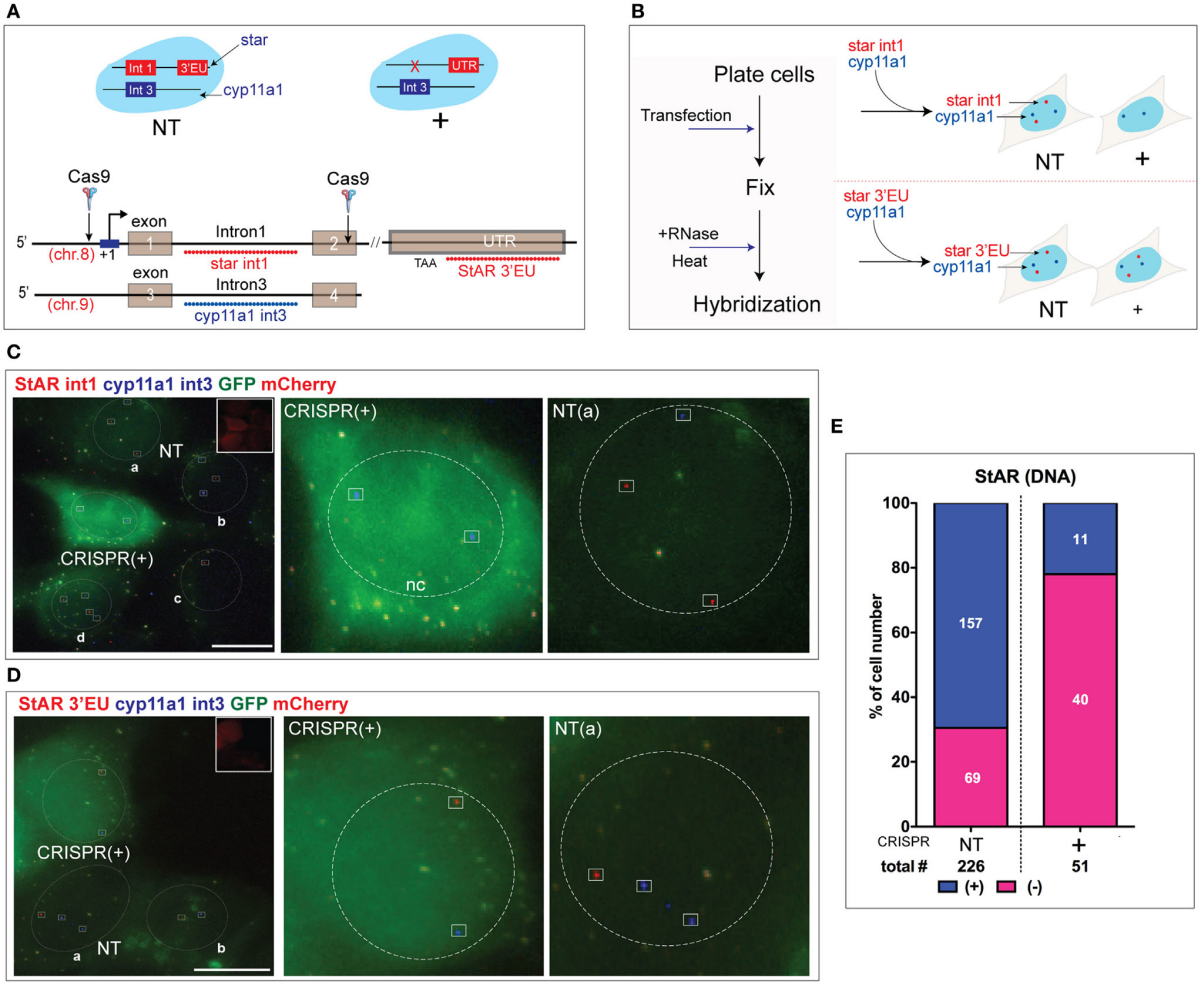
Detection of DNA deletion from the steroidogenic acute regulatory protein (STAR) loci of Y-1 cells by Cas9. **(A)** Probe design of DNA FISH. The probe hybridizes the StAR intron 1 (538/559 nm, pseudocolored red) in the CRISPR targeted sequence compared to non-target exon 7 [UTR/oligomer set targeting extended 3′UTR (3′EU), 538/559 nm, pseudocolored red] and Cyp11a1 int3 (618/637 nm, pseudocolored blue). **(B)** Procedure for DNA FISH. After RNase treatment, the slide was heated to denature DNA. Probes for CRISPR target StAR (int1) and non-target Cyp11a1 show the difference between NT and CRISPR (+) cells (top). Non-target StAR (3′EU) and Cyp11a1 were used as a control (bottom). **(C)** StAR intron1 was deleted in CRISPR (+) cells. StAR intron 1 compared to Cyp11a1 locus DNA retained in NT (a–d) cells. GFP and mCherry were used as markers for gRNA expression. The dashed lines (circle) indicate the nucleus. Blue loci/dots are Cyp11a1, and red loci/dots are StAR intron 1. Orange channel (pseudocolored red) was used for the detection of StAR int1 and StAR oligomer set targeting extended 3′UTR (3′EU). Red channel (pseudocolored blue) was used for Cyp11a1 int3. **(D)** Probes hybridizing CRISPR non-target sequences in StAR (3′EU) and cyp11a1 were applied in CRISPR (+) and NT cells. Red loci/dots are StAR exon 7. **(E)** Quantitation of StAR intron 1. The blue segment in the bar denotes the percentage of cells with the active locus (StAR p-RNA). Scale bar represents 10 µm.

To verify whether excision of the targeted sequence had occurred in individual loci of StAR, DNA sm-FISH was performed with the p-RNA probe, which hybridizes to intron 1 which is targeted by the dual gRNACRISPR/Cas9. We compared this hybridization to non-target DNA either within the StAR locus (distal promoter-3′EU/exon 7) or in Cyp11a1, another expressed gene (intron 3/Cyp11a1) (Figure 5A). The key steps in distinguishing DNA sm-FISH from RNA sm-FISH are the RNase removal of all RNA and the stronger denaturation conditions needed to open the chromatin structure for hybridization (Figure 5B). The gene hybridization targets a single DNA sequence at each locus whereas the RNA expression also detected by p-RNA may vary from 1 to >50 transcripts.

We compared StAR and Cyp11a1 DNA in two CRISPR (+) cells with four adjacent NT-cells. CRISPR (+) cells (#1 and #2) each showed a pair of Cyp11a1 loci but no StAR intron 1 (Figure 5C). Overall, these CRISPR (+) cells showed that about 20% retained at least 1 StAR locus that retained intron 1. Of the four adjacent NT cells, Cyp11a1 loci are seen in three with an average of one locus detected per cell. The NT cells each show at least the intron 1 DNA segment from StAR loci (Figure S5A in Supplementary Material). A similar heterogeneity in sm-FISH hybridization was seen in the Cyp11a1 transcription (Figure 3D). A similar probing with the StAR exon 7 DNA probe (3′EU) showed that StAR DNA outside the targeted segment was retained in the CRISPR (+) cells to the same extent as in the adjacent NT cells (Figure 5D; Figure S5B in Supplementary Material). Again the 3′EU sequence was detected at one locus in each of the CRISPR (+) cells. Thus, both StAR 3′EU DNA and Cyp11a1 shared similar detection efficiencies. The locus DNA hybridization that requires denaturation at a much higher temperature than the locus-associated RNA is likely to be easier for regions of the chromatin that are actively transcribed. A distal StAR promoter sequence with similar base composition requires still a harsher denaturation (Figure S5C in Supplementary Material).

An analysis of the StAR gene in 277 cells showed recognition of at least 1 StAR intron 1 sequence in 157/226 UT cells (70%) but in only 11/51 CRISPR (+) cells (20%) (Figure 5E). The CRISPR (+) cells that retain StAR intron 1 closely parallel the 25% that retain p-RNA and sp-RNA transcription without mRNA and protein.

### Time Course of CRISPR Initiation and StAR Editing in Y-1 Cells

**Figure 6:**
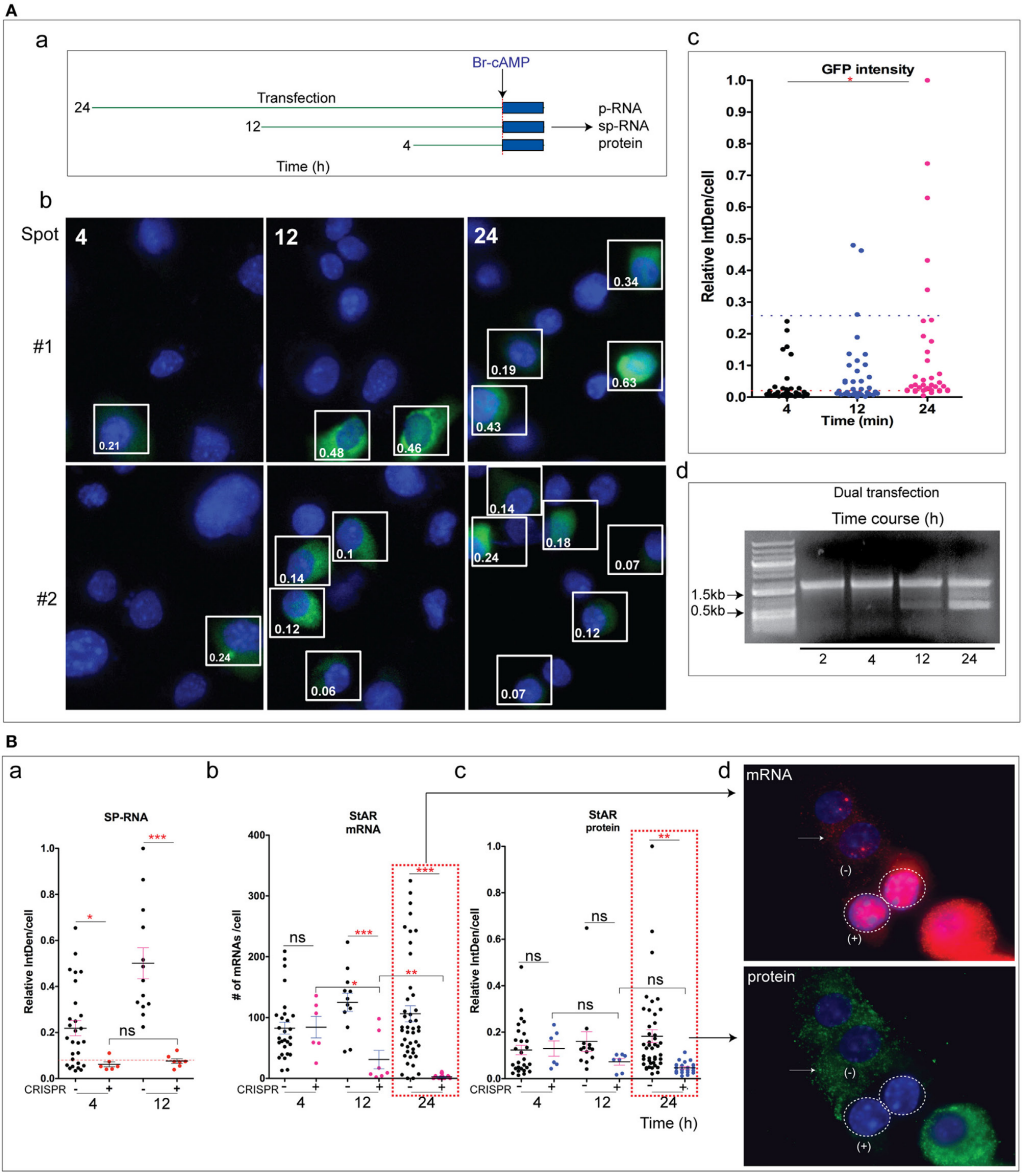
**(A)** Time course of steroidogenic acute regulatory protein (StAR) editing in relation to Cas9 transfection. (a) Design for varying post-transfection time for CRISPR editing: after the transfection period, Br-cAMP (1 mM) was treated for 2 h. p-RNA, sp-RNA/mRNA, and protein in CRISPR (+) and NT cells were analyzed by high-resolution fluorescence *in situ* hybridization. (b) Duplicate transfections (#1 and #2) were used to show GFP expression in cells as a function of post-transfection time (4, 12, and 24 h). (c) Relative GFP intensity in individual cells was shown. Two dotted lines separate three levels of expression: lower—background; middle—effective CRISPR (+); upper—effective but in excess of the amount needed for CRISPR. (d) PCR strategy to validate StAR gene deletion. Time course experiment was performed (2–24 h). **(B)** Impact of CRISPR on StAR editing at various transfection times: (a) StAR locus (sp-RNA), 4 versus 12 h; (b,c) loss of mRNA and protein, 4, 12, and 24 h. (d) The impact of CRISPR/Cas9 on StAR mRNA and protein. Error bars show SEM. *p < 0.05, **p < 0.01, ***p < 0.001; ns, not significant by ANOVA with *post hoc* Tukey. Scale bar represents 10 µm.

We have addressed the time dependence of several key steps: the entry of editing CRISPR vectors into the cells, their expression as marked by GFP and mCherry, the deletion of the targeted StAR DNA, and the several changes in StAR expression. We examined each at 4, 12, and 24 h after transfection. The editing responses were compared after a 2 h Br-cAMP stimulation [Figure 6A (a)]. Sm-FISH provides an assessment of the impact of increasing periods of CRISPR/Cas9 activity on StAR expression (locus sp-RNA versus cytoplasmic mRNA and protein). The gRNA/Cas9’s complex interacts with the StAR gene and generates the initial DSB within a few minutes of their expression, which is marked by synthesis of GFP and mCherry proteins. In this event, the Cas9/StAR editing is likely to occur on the leading edge of the GFP/RFP expression time profile. In Figure 5, we show that DNA hybridization in intron 1 remains heterogeneous in ways that may relate to the transcription access. The intensity of GFP fluorescence in each cell has been assessed for all cells in the fields of two CRISPR-transfected slide cultures at 4, 12, and 24 h [Figure 6A (b)]. We have used GFP fluorescence as the marker since mCherry expression overlapped in all cases. The GFP expression was robust in some cells after 4 h and approximately doubled in both numbers and intensities after 12 h. Between 12 and 24 h the number of cells expressing GFP remained at about 30%, but the mean expression has doubled. We presented three GFP expression ranges: low/background in which transfection is minimal, medium, which is sufficient to generate DSB and a higher level for which the editing impact is unknown [Figure 6A (c)].

We have used the PCR amplification of StAR DNA to analyze the effects of the single and dual CRISPR/Cas9 processes on the StAR locus (Figure 3C). We examined the excision as a time course in relation to the CRISPR/Cas9 GFP transfection [Figure 6A (d)]. After 12 h, there was about a 15–20% excision compared to the 50% excision after 24 h. However, after 4 h, the excision fragment was not detectable despite CRISPR/Cas9 transfection at about half the 12 h level. The 0.65 kb product should be readily detectable at 10% of the 24 h level representing 5% excision. Overall, the excision process matched the intensity of CRISPR/Cas9 transfection.

We examined the effects of editing in CRISPR (+) cells at the three times after CRISPR transfection followed by a 2 h Br-cAMP stimulation. Threefold increases in sp-RNA/mRNA in Y-1 cells produced by Br-cAMP after 2 h (Figure 2C) matched the increase in cytoplasmic mRNA (Figure 2D) and previous assessments of this response (1, 33). Thus, at least 75% of the RNA at the end of each 2 h Br-cAMP treatment was produced within this period and *after* the CRISPR–Cas9 transfection.

When the stimulation was initiated after 4 h, we saw the complete loss of locus sp-RNA expression from CRISPR (+) cells [Figure 6B (a)]. Surprisingly, mRNA expression was retained (Figure S6 in Supplementary Material). After 12 h, the suppression of locus sp-RNA continued, but there was now a modest decline in StAR mRNA and protein [Figure 6B (b–d)]. However, 24 h after transfection, there was the complete removal of StAR mRNA and protein in CRISPR (+) cells. The decline in StAR protein follows the loss of mRNA. The NT cells again showed modestly progressive increases.

The more rapid effect on the expression at the loci compared to the mRNA and protein indicated an editing mechanism that discriminates between locus expression and mRNA generation. The complete losses of locus RNA, mRNA, and protein at 24 h match the measured DNA deletion shown in Figure 6A (d). The diminished effects on mRNA and protein at 4 and 12 h correspond well with the diminished DNA deletion at these earlier times. The extensive suppression of sp-RNA at 4 and 12 h far exceed the DNA deletion at these times and therefore arise from another effect of the CRISPR/Cas9. As noted earlier, the single 5′gRNA CRISPR/Cas9 can impact StAR without any deletion.

### LDs Accumulate in CRISPR (+) Y-1 Cells but Only after StAR mRNA Deletion

**Figure 7:**
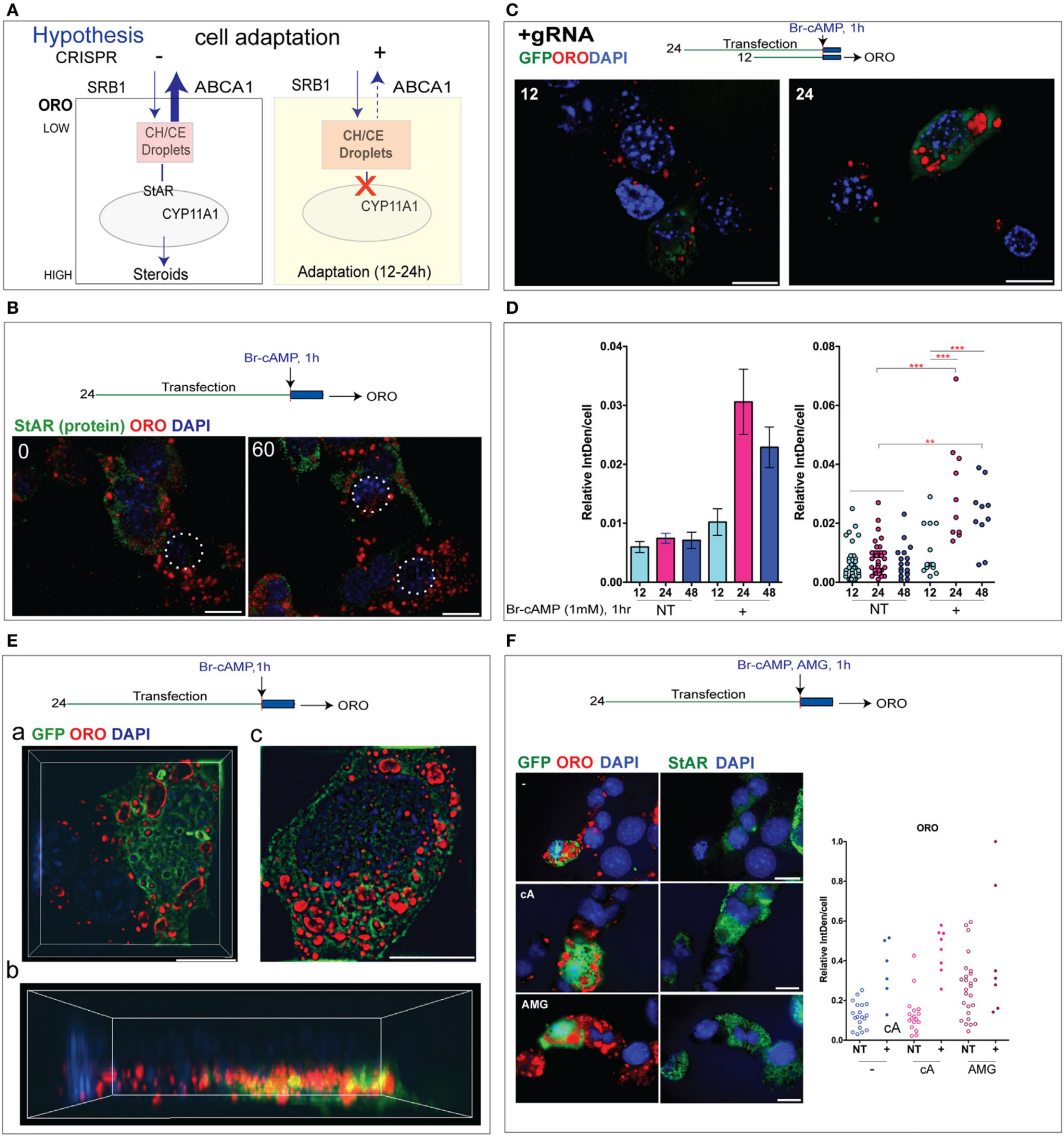
Cholesterol esters (CEs) in lipid droplets (LDs) appear selectively and rapidly in cells with CRISPR steroidogenic acute regulatory protein (StAR) deletion. **(A)** Model for StAR cytoplasmic activity. Protein kinase A activates CE conversion to cholesterol (CH) mediated by the hormone-sensitive lipase; transfer into the mitochondria-mediated by StAR leading to CYP-mediated metabolism (Cyp11a1 > Cyp27a1). StAR may also impact cholesterol uptake into the cell mediated by the high-density lipoprotein (HDL) receptor Srb1; internal transfer in and out of LDs and exit from the cell due to an ATP-dependent pump activity (Abca1, Abcg1). StAR deletion increases CE-containing LDs visualized and quantified through Oil Red O (ORO) staining. **(B)** CE in LDs measured by ORO in Y-1 cells after 24 h of CRISPR transfection separated from StAR protein expression in NT cells. CRISPR (+) cells are circled. **(C)** ORO staining in Y-1 cells at 12 and 24 h after the CRISPR transfection. **(D)** Quantitation of ORO intensity in individual cells at 12–48 h after transfection in Y-1 cells. **(E)** N-SIM images of GFP expression and ORO droplets in Y-1 cells. (a) XY section of CRISPR (+) cells expressing GFP and showing high ORO and an adjacent NT cell with typical low ORO. (b) The 3D assemblage of the same cell (c) different CRISPR cell with more extensive ORO staining but heterogeneous distribution. **(F)** Distribution of ORO and StAR in NT cells under basal conditions and after 1 h stimulation by Br-cAMP or aminoglutethimide. Left and right panels show same cells marked by, respectively, GFP and ORO (left) and StAR protein. In each case, ORO several large LDs locate together and displace StAR/mitochondria.

We cannot directly image the effect of StAR on steroid synthesis, but LDs, which can be readily imaged by ORO, may provide an alternative target (19). In steroidogenic cells, these droplets mostly comprise CE. The CE content of LDs reflects the complex cholesterol dynamics of the cytoplasm, which involves transport through endosomes, biosynthesis from acetyl-CoA, and acylation by cholesterol acyltransferase (34). LDs include some endosomes that are filled with CE derived from low-density lipoprotein (LDL) but in rodent cells mostly comprise CE droplets that derive from high-density lipoprotein (HDL) taken up by Srb1. These droplets are surrounded by organizing proteins that also control interactions with processing enzymes such as HSL and StAR (18). A change of CE in LDs in CRISPR (+) cells visualized by ORO may, therefore, represent an adaptation to StAR deletion CRISPR (+) (Figure 7A).

There appears to be an inverse relationship between the levels and location of ORO (+) droplets and the mitochondria that express StAR protein. Y-1 cells with high StAR have low ORO staining, and this inverse relationship extends to the cells (Figure 7B). We have shown that CRISPR (+) cells that are marked by GFP show substantial increases in ORO, which start 12 h after CRISPR transfection (Figure 7C). To assess this adaptation to the Cas9 editing of StAR, we examined ORO staining in Y-1 cells at 12, 24, and 48 h after the CRISPR transfection. Each time, Br-cAMP was added for the final 1 h to match the conditions used for the assessment of StAR editing. A comparison of ORO staining in CRISPR (+) and adjacent NT cells shows a large increase in CE/ORO in the CRISPR (+) cells that lack StAR activity (Figure 7D).

A high-resolution N-SIM image of ORO in adjacent NT and CRISPR (+) cells showed CE/ORO lining the inside of vacuoles [Figure 7E (a)]. We further examined the NT/CRISPR (+) pair [Figure 7E (a)] with a 3D compilation [Figure 7E (b)]. For the most part, the ORO accumulations are separate from the GFP accumulations and are positioned above them. There are also many vacuoles lined with GFP. The second set of three CRISPR (+) cells [Figure 7E (c)] showed appreciable local heterogeneity in the stimulation of ORO (+) CE accumulations. There was little difference in the accumulation of CE regardless of the presence or absence of Br-cAMP, which activates both StAR and HSL. The regions of high ORO staining in NT cells again avoided regions of StAR expression in these cells [Figure 7F (a)]. We also tested the effect of a 1 h addition of the Cyp11a1 inhibitor aminoglutethimide (AMG), which fully inhibits mitochondrial cholesterol metabolism (39). AMG also increased ORO staining in NT cells in regions devoid of StAR. After the AMG treatment, 60% of the basal cells showed increased ORO with enlarged LDs or CE-laden endosomes, much like what was seen in StAR deficiency in CRISPR (+) cells. The AMG treatment of CRISPR (+) cells produced additive increases in ORO [Figure 7F (b)]. The effect of AMG is remarkably rapid, raising the question of whether AMG is affecting CE distribution at an additional point.

The relationship between ORO and StAR editing remains similar to the plot for GFP accumulation. The accumulation of GFP in vacuoles raised a possibility of interference with CE trafficking. To check this possibility, we followed the conditions shown in Figure 4C and omitted both the gRNAs that are necessary for the Cas9 cleavage of StAR without affecting GFP expression. We measured the ORO changes in NT and GFP (+) cells. Without gRNA to initiate StAR editing, the GFP (+) cells showed no stimulation of ORO staining to match the high cytoplasmic GFP expression [Figure S7A in Supplementary Material]. This control was confirmed for multiple gRNA (−) cells [Figure S7B in Supplementary Material]. The significant increase in CE accumulations, therefore, directly derives within a few hours from the redirection of cytoplasmic cholesterol dynamics resulting from the removal of StAR.

## Discussion

This report describes the direct imaging with sm-FISH of the dual-gRNA-directed CRISPR/Cas9 inactivation of the key steroidogenic gatekeeper gene StAR. PCR analyses showed the excision of a central segment of proximal promoter/exon 1/intron 1 from the StAR genomic DNA. In addition, sm-FISH provided comprehensive single-cell imaging of CRISPR editing at the level of primary and spliced transcripts at gene loci and single molecules of cytoplasmic mRNA in relation to expressed protein and gene function. The sm-FISH method has also been extended to provide novel direct single-cell imaging of the DNA deletion in CRISPR (+) cells and adjacent NT cells. The extent of this StAR deletion is compared to retentions of, respectively, a non-targeted segment of the StAR gene and the Cyp11a1 gene, the immediate partner in the steroidogenic pathway. We applied this dual gRNA Cas9 targeting method to the StAR gene in Y-1 adrenal cells and MA-10 interstitial testis cells, each of which showed an acute PKA stimulation, thus providing extra insight into CRISPR’s impact. This strategy, which is applicable to any responsive gene, provides novel direct assessment of the immediate impact of CRISPR editing on both the gene expression mechanism and the function.

We marked CRISPR (+) cells using mCherry and GFP fluorescence (Figures 2, 4 and 6), which additionally provides a time course for effective transfection (Figure 6). PCR shows that half of the StAR DNA exhibited the predicted 1.1 kb deletion 24 h after transfection (Figure 6), while sm-FISH shows a complete loss of StAR expression of mRNA and protein from CRISPR (+) cells (Figure 3). This loss was matched by excisions of the dual CRISPR target sequence as shown by sm-FISH assessment of intron 1 (Figure 5). This loss of StAR expression was matched by a large increase in CE accumulation in LDs and endosomes, which we imaged using ORO. We also observed this accumulation in humans with StAR deficiency and in StAR^−/−^ mice (40).

However, in addition to excision, 30% of the CRISPR (+) cells retained this StAR sequence and normal p-RNA and sp-RNA transcripts at the loci, but remarkably, lost StAR mRNA and protein in the cytoplasm (Figure 3). We detected similar losses when using a single 5′gRNA (but not a 3′gRNA) (Figure S5 in Supplementary Material), thus establishing that alternative mutation-based mechanisms can operate in parallel with DNA excision.

Y-1 cells exhibit modest basal StAR expression, which is sufficient to sustain maximum cholesterol metabolism when activated by Br-cAMP (7). This expression is diversely distributed among the individual cells and their loci [Figure 2 (b)]. The impact on basal expression in CRISPR (+) cells is consistent with deletion but currently, lacks a sufficient number of images from more active basal cells to achieve a statistical assessment. We overcame this difficulty by applying CRISPR/Cas9 before the appreciable induction by Br-cAMP, when most cells exhibit strong locus and cytoplasmic responses (Figures 2 and 3). The precision of the sm-FISH in resolving transcription and splicing at the locus from processing to mRNA established distinct early (4 h) and delayed (12–24 h) alternative disruptions of the StAR locus without deletion that nevertheless retained transcription. Sm-FISH analyses of MA-10 cells differ in that they lack measurable basal locus and cytoplasm expression. This distinction facilitated our analysis of the complex StAR response to Br-cAMP stimulation (Figure 4; Figure S4 in Supplementary Material). The time course for CRISPR/Cas9 effects showed that editing was effective within 4 h in removing StAR expression at the locus, even though mRNA and protein were retained. Examination of single 5′end targeting was selectively effective within 24 h in suppressing StAR expression without DNA deletion suggesting that this 4 h disruption may arise from an error in the early DSB repair of the 5′gRNA Cas9 process in the proximal promoter. Alternatively, direct inhibition of StAR transcription factors such as CREB or C/EBPβ may be produced by this high affinity Cas9 complex. Removal of this complex through the slow mutation will remove this direct disruption.

These two alternative CRISPR editing effects were consistent with selective interventions in two StAR multistep expression processes that functioned in parallel after 2 h of Br-cAMP stimulation of both Y-1 or MA-10 cells (1, 33) (Figure S8 in Supplementary Material). StAR loci are initially activated with substantial restraints over splicing and possibly polyadenylation such that p-RNA and sp-RNA build up at the loci with the only slow transfer of mRNA to the cytoplasm. Protein translation, however, was robust despite this low cytoplasmic mRNA. This early stimulus corresponded to maximum cholesterol metabolism. This activity depends on the translation of a labile 3.5 kb mRNA that locates to mitochondria. This location depends on the extended 3′UTR of StAR, which delivers cleavage and polyadenylation at specific sites (30). After about 1 h, a much faster transcription process began in which formation of p-RNA at loci is bypassed through a coupling of splicing to transcription. We suspect that this surge derived from Br-cAMP induction of additional StAR transcription factors, notably c/EBPβ and NR4a1 (6, 41). The rapid sp-RNA processing meant that sp-RNA remained low in the loci while mRNA accumulated extensively in the cytoplasm, particularly at the mitochondria. At the end of a 2-h Br-cAMP stimulus, both transcription mechanisms were functioning equally.

The 4-h CRISPR effect was consistent with disruption of the uncoupled process, which provided locus RNA but contributed little to mRNA. The slower 12–24 h alternative editing without excision targeted the rapid coupled process, which did not contribute to locus p-RNA or sp-RNA but generated most of the StAR mRNA and protein. This alternative to CRISPR-induced excision implies a distinct slower Cas9 intervention in the StAR promoter that may arise as an error in the excision ligation step (Figure S8B in Supplementary Material). The equivalent effects of the single 5′gRNA CRISPR/Cas9 strategy established that a single promoter intervention proximal to the 5′PAM site can produce such changes, presumably from mutations derived from errors in the initial NHEJ-type repair process (42). Interestingly, the endonuclease cleavage site overlapped the C/EBPβ-binding sequence, which likely mediated of the delayed coupled StAR transcription (41).

The CRISPR editing machinery appears to function more rapidly than expected based on the *in vitro* modeling (43). However, a novel recent approach to the measurement of the dissociation of gRNA/Cas9 complexes in cells has indicated much faster rates (44). These cell dissociation rates vary appreciably, probably because of different competing binding effects from transcription factors. Here, the 5′gRNA interaction in the StAR proximal promoter region overlapped several functional transcription factor sites.

**Figure 8:**
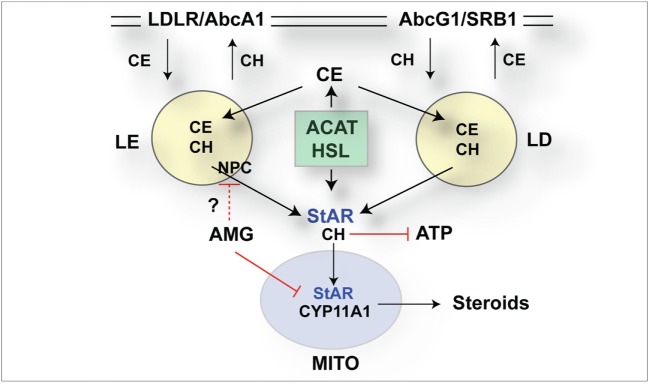
Schematic diagrams of selective CRISPR/Cas9 impact on synthesis of steroidogenic acute regulatory protein (StAR) mRNA and cholesterol transfer activity in the cytoplasm. CRISPR/Cas9 editing of StAR increases accumulation of cholesterol esters (CEs) detected by Oil Red O (ORO). Newly translated p37 StAR becomes phosphorylated by protein kinase A and then imported into mitochondria concomitant with cholesterol (CH) transfer and metabolism by Cyp11a1 (52). Without phosphorylation, StAR may promote cytoplasmic steps that promote exit of excess CH from the cells; for example by stimulation of liver X receptor (LXR) induction of ATP-dependent cholesterol export pumps: AbcA1 to low-density lipoprotein (LDL) and AbcG1 to high-density lipoprotein (HDL) (21). Entry from LDL deposits cholesterol in the late endosomes/lysosomes. Re-distribution, including endoplasmic reticulum or mitochondria, is directed by NPC1 and 2 together with StARD3/MLN64 (48). Entry from HDL deposits CE in LDs. CE is converted to CH by hormone-sensitive lipase (HSL) and in LE by acid-sensitive esterase (18, 53). Acylation by cholesterol acyltransferase (ACAT) produces the reverse formation of CE. StAR inactivation by Cas9 increases CE. This can arise through decreased removal from LE and LD and decreased export from the cell *via* AbcA1 and AbcG1. Aminoglutethimide (AMG) and StAR/Cas9 share similar effects on ORO/CE. Decreased CH metabolism cannot produce AMG effect (1 h). Other points of overlap include effects on mitochondrial cholesterol that reduce ATP, or decreased distribution of CE from LE. Lipophilic amines like AMG, notably imipramine, disrupt the release of CH from LE (54–56). StAR may also mediate the activation of LXR, notably to maintain AbcA1-mediated CH transport.

The role of StAR as a regulator of cholesterol metabolism in mitochondria has been extensively documented (7, 45). Here, the CRISPR/Cas9 removal of the StAR activity between 12 and 24 h has been linked to a large increase in CE in LDs (Figure 7), which StAR affects through interactions with a HSL and activation of alternative transcription processes mediated by LXRa (22, 46). This accumulation in single cells imaged by ORO staining provides a measure of the cell adaptation to StAR loss (Figure 7A). Increases in CE in LD and late endosomes in these cells were enriched in regions where StAR protein and associated mitochondria were low (Figure 7). The Cyp11a1/cholesterol metabolism inhibitor AMG (39) rapidly increased these effects in NT cells independent of StAR, making the involvement of cholesterol metabolism unlikely. Each ORO image following StAR deletion in CRISPR (+) cells and AMG stimulation in NT cells showed similar accumulations in cytoplasmic endosome-like vacuoles (Figure 7E). CE in LDs is supplied by HDL *via* SRB1 at the plasma membrane but in late endosomes by LDL through LDL receptors. Each import is balanced by ATP-dependent export pumps (Abcg1 and Abca1). Potential mechanisms include stimulation of ATP-dependent cholesterol export (47), mitochondrial ATP and apoptotic processes (46), and the effects of hydroxyl-cholesterol metabolites on LXR receptors (21). These cytoplasmic disruptions are represented in relation to CRISPR editing (Figure 8). StAR deletion appears to affect enhance the complex cytoplasmic cholesterol exchange dynamics involving cholesterol movement between multiple organelles. Interestingly, NPC/STARD3 mediates removal from late endosomes/lysosomes (LE/Ly), which are inhibited by lipophilic amines (48) that may potentially include AMG. NPC1/Stard3 mediates contacts between LE/Ly and endoplasmic reticulum (ER), which mediate cholesterol transfer between these organelles (49, 50). ER contacts with mitochondria facilitate their fusion, which is now recognized as a prime site of StAR activity (51). This rapid response to dual CRISPR Cas9 editing of StAR closely matched the cytoplasmic large CE accumulations in the adrenals, testes, and ovaries of StAR^−/−^ mice, as well as in humans, who carry the loss of function StAR mutations (40).

In this report, we emphasize the effectiveness of sm-FISH in the analysis of CRISPR/Cas9 editing. This approach provides the means to identify alternative variations, for example, changing the gRNA sites while also providing insight into the optimum conditions for more detailed single-cell cloning or sequencing.

## Author Contributions

JL and CJ designed and analyzed the experiments; wrote the manuscript. JL carried out all experiments.

## Conflict of Interest Statement

The authors declare that the research was conducted in the absence of any commercial or financial relationships that could be construed as a potential conflict of interest.

## Footnotes

^1^http://tools.genome-engineering.org.

^2^http://chopchop.cbu.uib.no.

^3^http://www.rgenome.net/cas-offinder/.

^4^http://www.biosearchtech.com/products/rna-fish

^5^http://www.rajlab.seas.upenn.edu/StarSearch/launch.html.

## Abbreviations

3′EU, oligomer set targeting extended 3′UTR; Cas9, CRISPR-associated protein 9; CE, cholesterol ester; CRISPR, the clustered regularly interspaced short palindromic repeat; DSB, double-strand breaks; FISH, fluorescence *in situ* hybridization; gRNA, guide RNA; HSL, hormone-sensitive lipase; LD, lipid droplets; N-SIM, Nikon’s Structured Illumination Microscope; PAM, protospacer adjacent motif; PKA, protein kinase A; p-RNA, primary RNA; Single Molecule Fluorescence in Situ Hybridization (sm-FISH); sp-RNA, spliced primary RNA; StAR, steroidogenic acute regulatory protein.
