# Supplementary material for: Monitoring of Dual CRISPR/Cas9-Mediated Steroidogenic Acute Regulatory Protein Gene Deletion and Cholesterol Accumulation Using High-Resolution Fluorescence In Situ Hybridization in a Single Cell
Source: Front Endocrinol (Lausanne). 2017 Oct 25;8:289. doi: 10.3389/fendo.2017.00289 (PMC5660980; doi:10.3389/fendo.2017.00289)
Supplement: Supplementary file 1 [file Image_1.PDF]

Supplemental figures

Monitoring of dual CRISPR/Cas9 mediated StAR gene deletion and cholesterol accumulation using high-resolution fluorescence in situ hybridization in a single cell

Jinwoo Lee, [a,b,*] and Colin Jefcoate [a,b,c,d,*]

*Corresponding Authors:
Jinwoo Lee
Colin Jefcoate

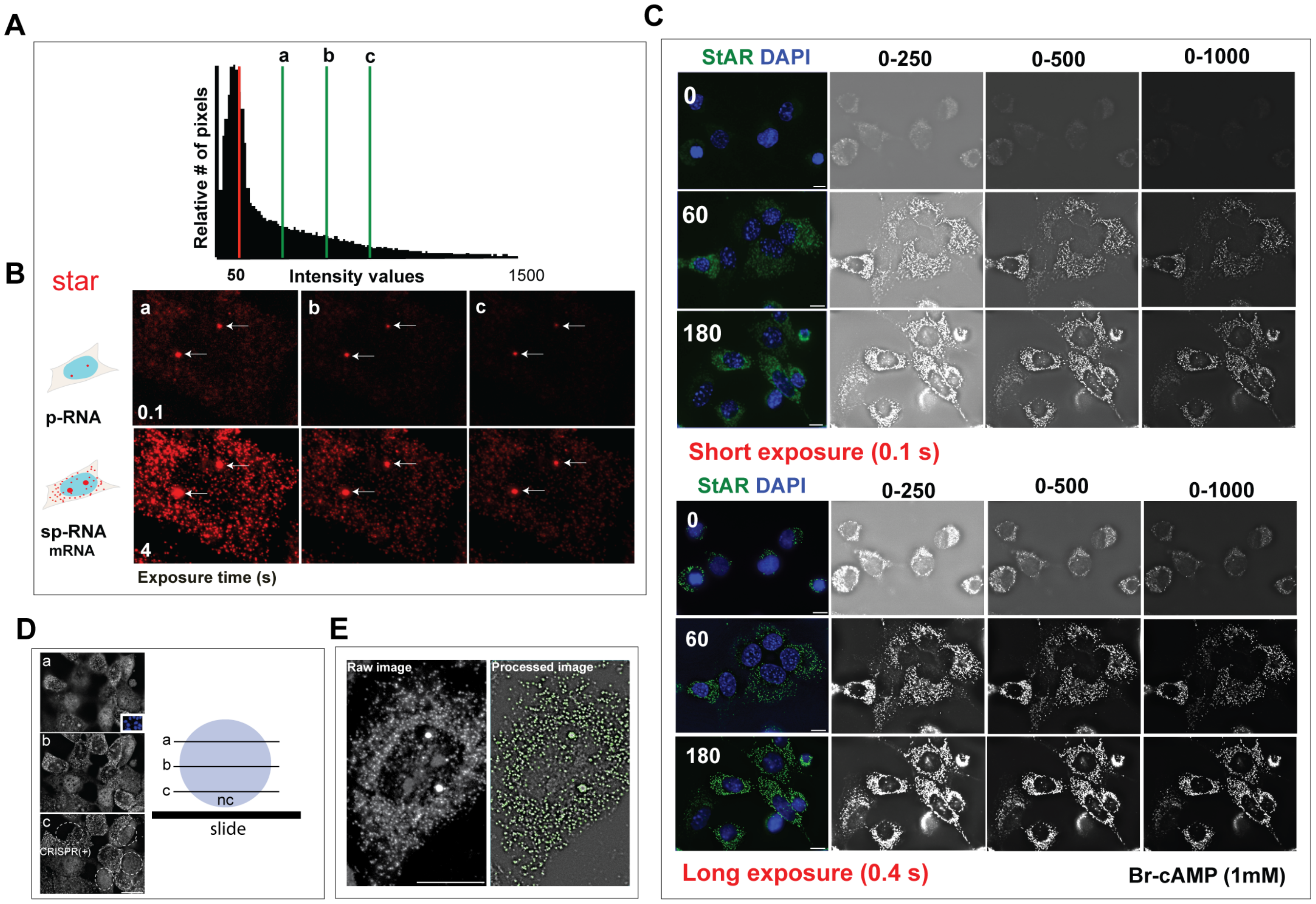

Figure S1. Image analysis (sensitivity variation)

A. Pixels versus intensity. The Y-axis indicates the relative number of pixels, and the X-axis shows the intensity values. The red line describes the background value. The green lines show the data values across the entire image. B. sp-RNA images of Y-1 loci RNA and cytoplasmic mRNA after 180 min. of stimulation of Y-1 cells by Br-cAMP. These cells (top and bottom panel) are viewed at different exposure times. Loci and mRNA have different data values, so the exposure time and intensity values should be optimized. To avoid saturation, a short exposure time (IX81: 0.1s) is necessary for loci detection. On the other hand, mRNA particles have the lower mass, so they require longer exposures (IX81: 4s). C. StAR protein images at different exposure times and settings. The basal expression is barely detectable after 0.1 sec. Exposures but is visible after a 0.4 sec. exposure. However, the difference between basal and 180 min. Stimulation is minimized (pixel saturation). Short exposures show the true difference in responses to stimulation. Scale bar represents 10μm. D. Sliced XY images (a, b, c) were taken across z-axis. Ten cells including CRISPR (+) and NT cells viewed at three different XY

planes. Most mRNA resides in c plane, which is close to the bottom, so c was chosen for the calculation. E. The image of the bottom section shows removal of mRNA from CRISPR (+) cells. Processed image for mRNA counting with “STAR search” software. Scale bar represents 10μm.

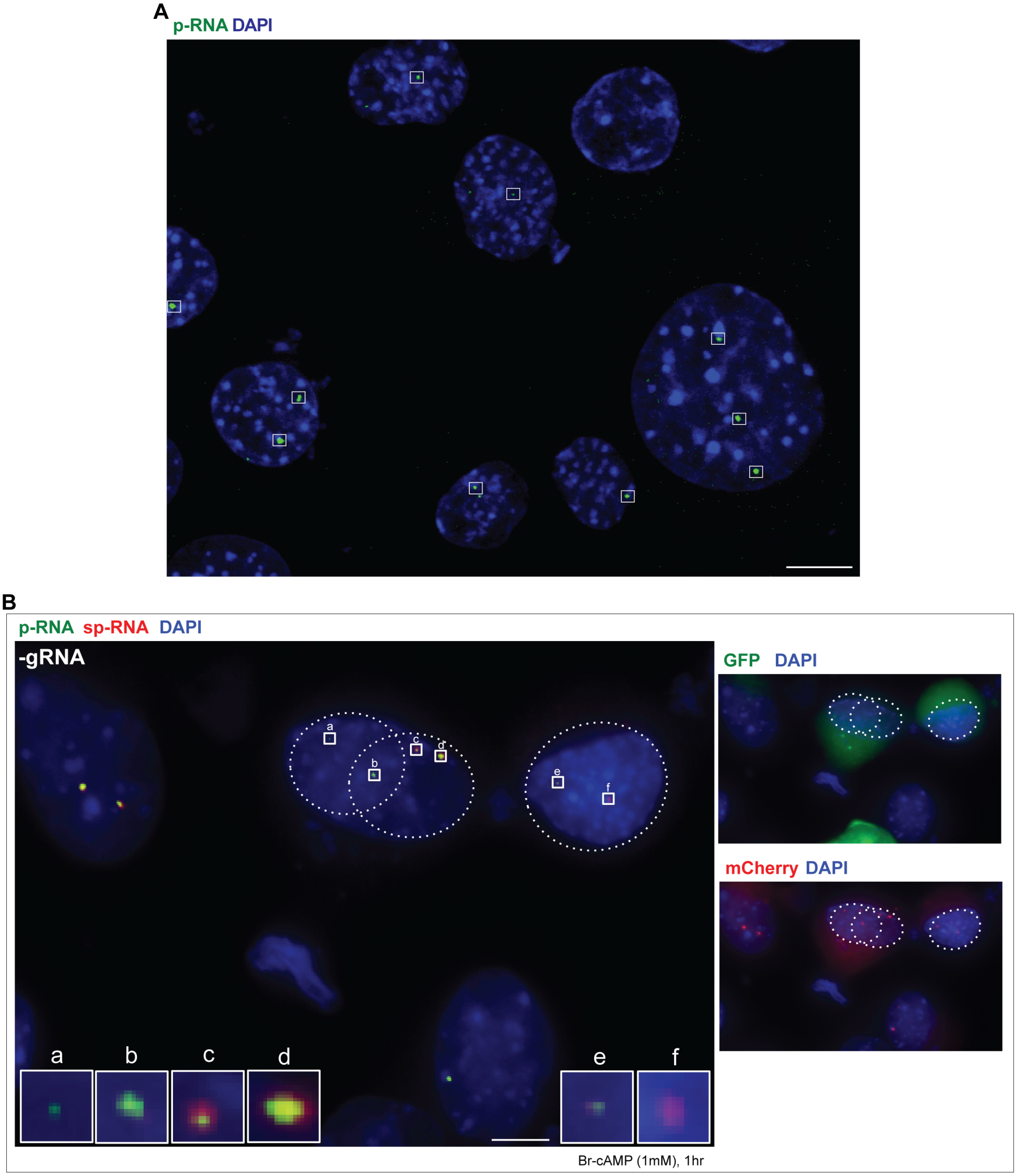

Figure S2. Imaging basal p-RNA

A. Basal p-RNA at low sensitivity (0.1 sec) (also Figure 3A (a)) compared to p-RNA image at high sensitivity (0.4 sec). B. Y-1 cells imaged with p-RNA and sp-RNA after CRISPR transfection (24 h) without gRNA. Scale bar represents 10μm.

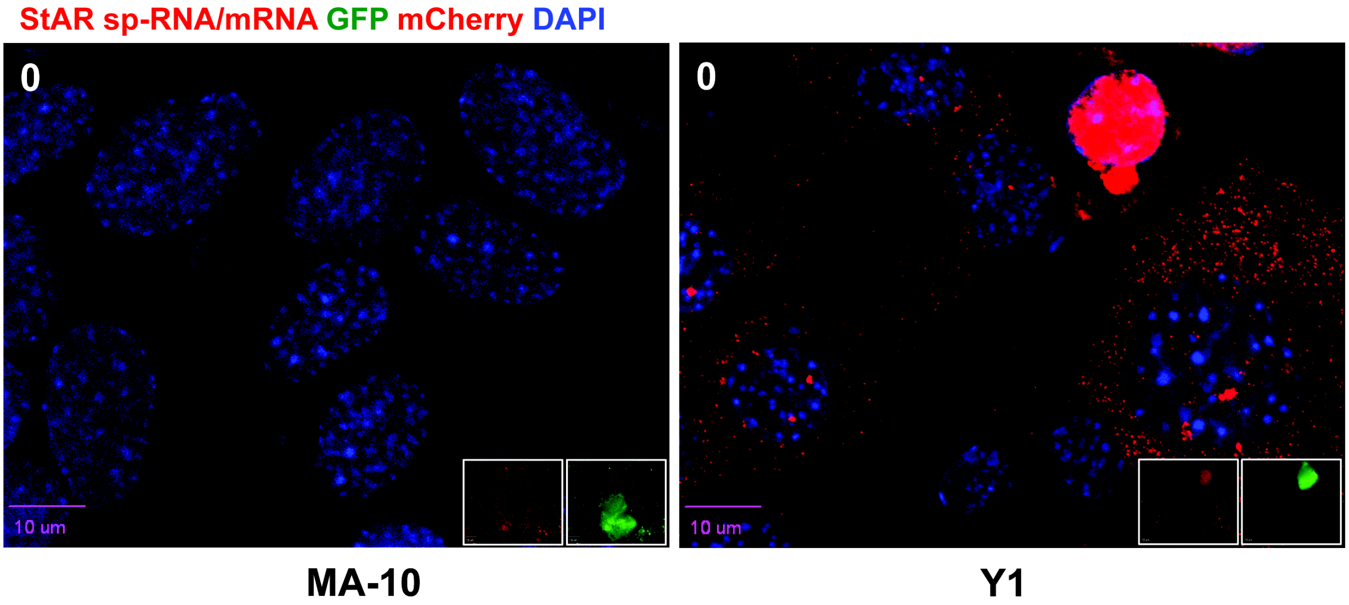

Figure S3. Basal MA10 cells compared to Y-1 cells at high sensitivity

MA10 cells and Y-1 cells (see Fig 3A) compared for basal expression using sp-RNA probes at high sensitivity. Scale bar represents 10μm.

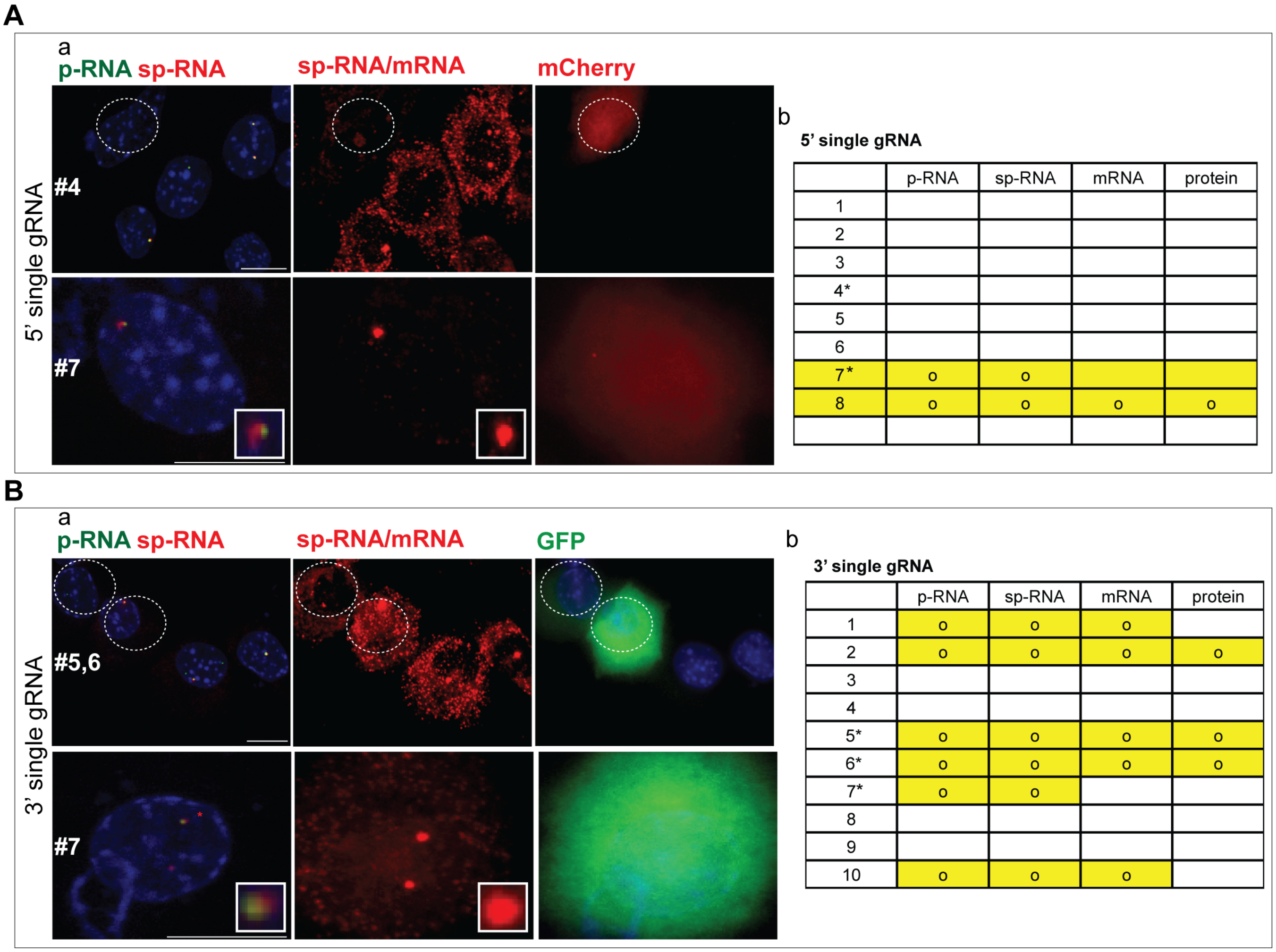

Figure S4. Single 5' (target: promoter)- and 3' (target: exon2) gRNA

A. (a) 5' gRNA CRISPR (+) /RFP (+) cells from #4 and cell #7 on the right panel. (b) O indicates that it is visible. B. 3' gRNA CRISPR (+)/GFP cells from # 5 and 6. C. Dual gRNA CRISPR (+) cells that retain transcription --# 4,5,6. Scale bar represents 10μm.

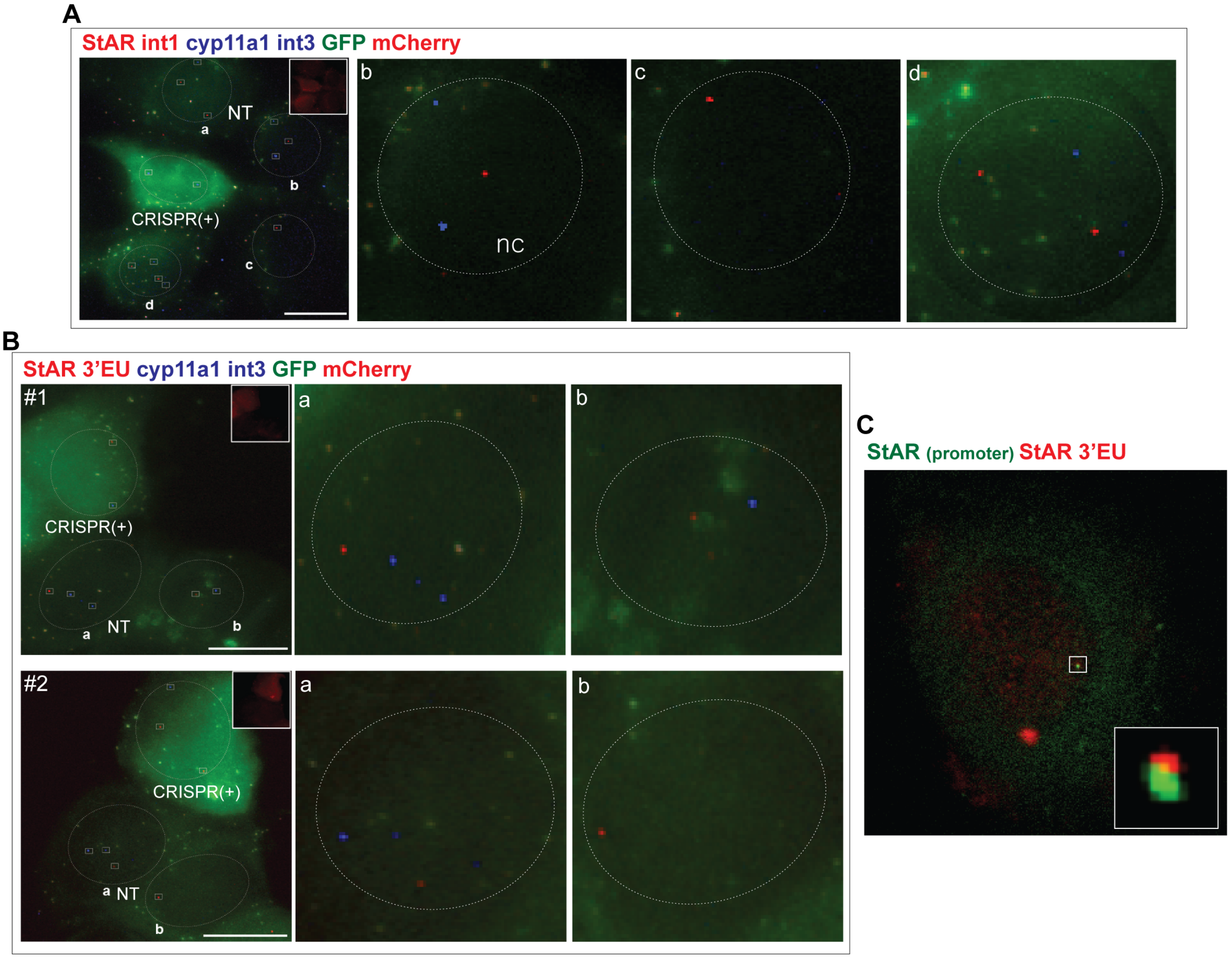

Figure S5. StAR and Cyp11a1 loci in non-transfected Y-1 cells (compare Figure 5)

A. StAR intron 1 compared to non-target Cyp11a1 intron 3 in 4 NT cells. B. Non-target StAR exon 7 compared to Cyp11a1. C. DNA FISH of distal StAR promoter and StAR 3'EU imaged in NT cells. Scale bar represents 10μm.

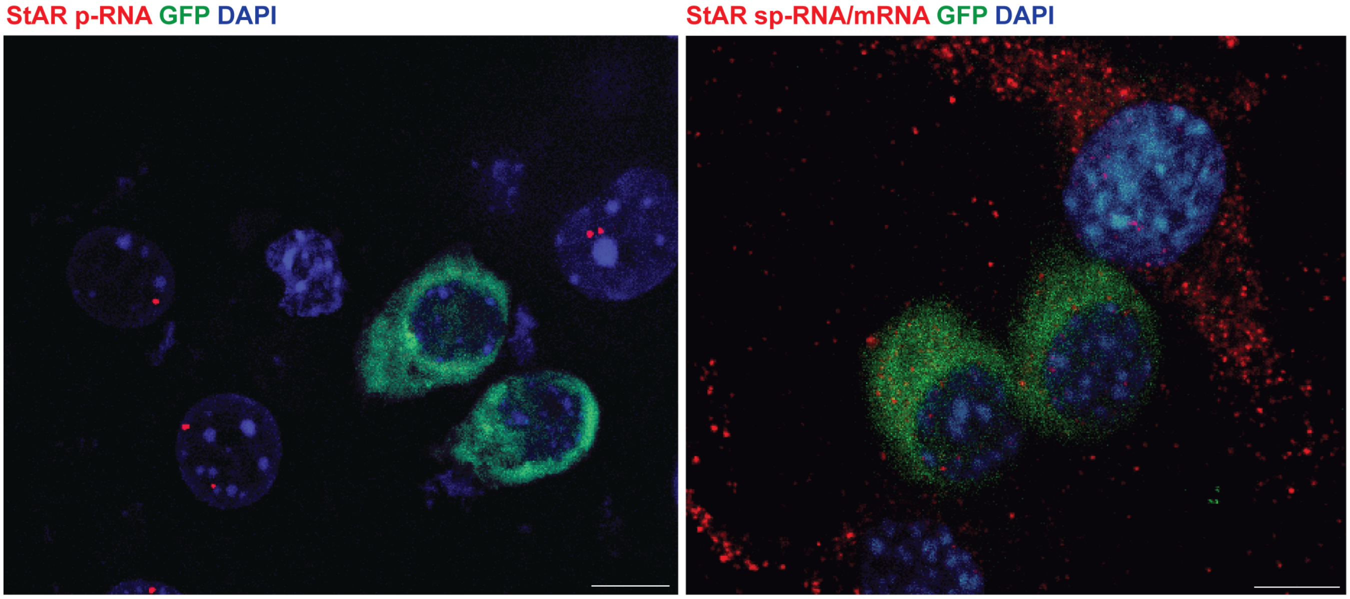

Figure S6. CRISPR (+) editing after 4 hours

CRISPR (+) editing of StAR after 4h, which removes transcription of loose Br-cAMP-induced p-RNA transcripts (left), not sp-RNA/mRNA (right). Scale bar represents 10μm.

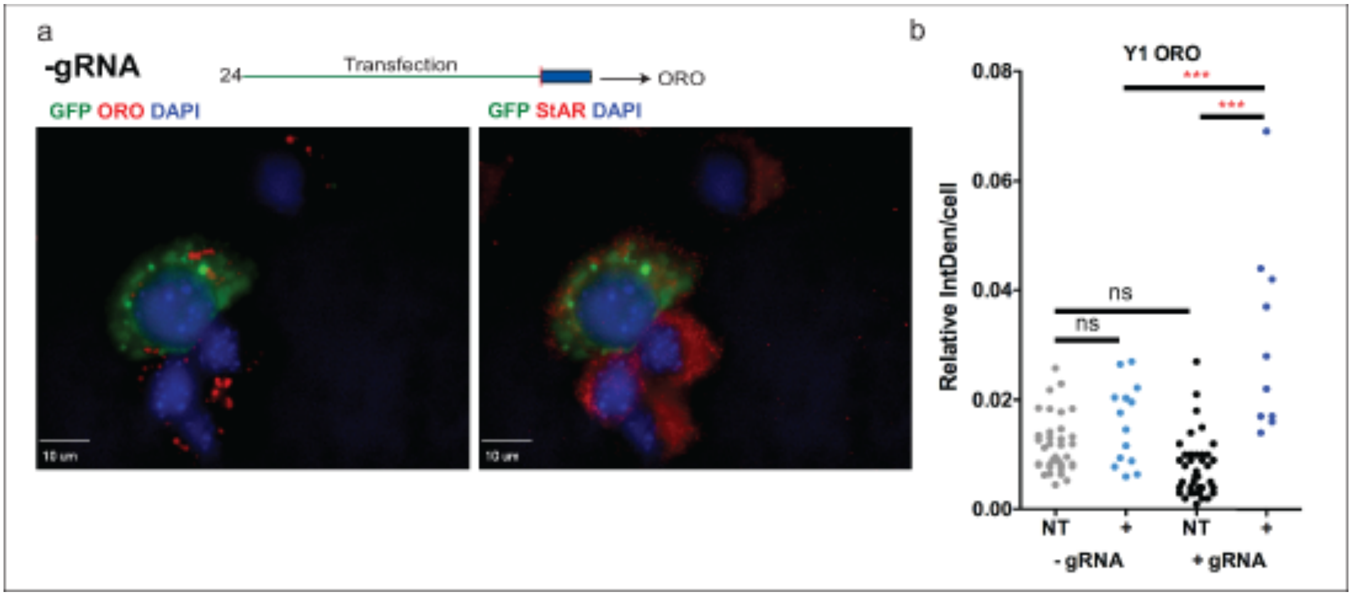

Figure S7. Removal of gRNA and Cas9 activity prevents lipid droplet increases. CRISPR transfection is carried out without gRNA, thus producing GFP and Cas9 without the targeted editing of StAR. A. There is no accumulation of ORO, as shown in representative GFP (+) cells. B. ORO analysis of all GFP cells in a field in comparison to adjacent NT cells. Scale bar represents 10μm. Error bars show SEM. *$p<0.05$, **$p < 0.01$, ***$p < 0.001$; ns, not significant by ANOVA with post-hoc Tukey.

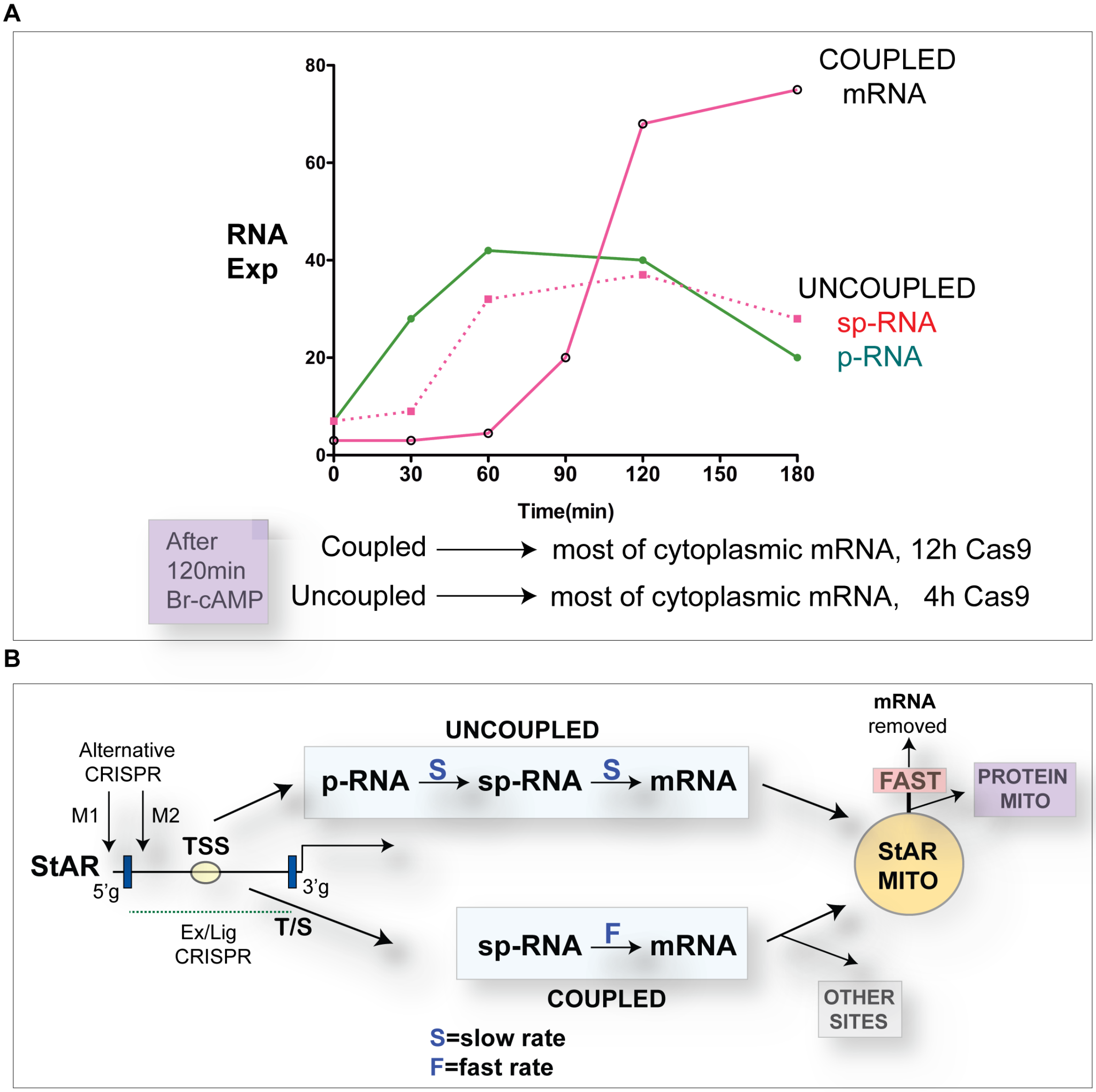

Figure S8. Schematic diagrams of selective CRISPR/Cas9 impact on synthesis of StAR mRNA

A. Separation of B-cAMP stimulation of StAR transcription into early onset uncoupled mechanism and coupled late onset mechanism. Coupled mechanism derives from the enhanced presence of splicesome complex and enhanced transcription by inducible factors (NR4a1, C/EBPβ). Data corresponding to this scheme is shown (Figure 2). The coupled and uncoupled transcription are equally represented in Y-1 and MA10 cells after 120min of Br-cAMP stimulation. The uncoupled mechanism occurs in cells in which the splicing complex is poorly engaged with StAR loci. As a consequence, primary transcription generates p-RNA

without spliced sp-RNA. After 20-30 minutes splicing starts and sp-RNA appears at loci. After 1h, p-RNA reaches a steady state as transcription is balanced by splicing. Sp-RNA accumulates at the loci as polyadenylation to mRNA is also slow. Transfer of mRNA to the cytoplasm is slow and scarcely visible in the face of rapid turnover. This mRNA is however rapidly translated at the mitochondria in the process of delivering active phospho-StAR, which activates cholesterol transport into the mitochondria. Once imported to the mitochondrial matrix StAR protein turns over slowly and is therefore for more readily detected than mRNA. This uncoupled process may be selectively targeted within 4h by a mutation in the proximal promoter to the 5' PAM site. Between 60 and 90 minutes, transcription increases with the formation of mRNA in the cytoplasm but without any effect on the p-RNA steady state. This transition is indicative of the initiation of a separate transcription process, which exhibits rapid splicing, which we refer to as 'coupled' expression. Sp-RNA at the loci also increases little due to rapid polyadenylation to mRNA and an accompanying surge in transport to the cytoplasm. Translation produces parallel increases in StAR protein. Suppression of this coupled StAR expression accounts for the loss of mRNA and protein after 24 h. Retention of locus p-RNA and sp-RNA derives from the parallel uncoupled process, which is now unaffected. Deletion of StAR DNA removes both mechanisms.

B. Selective targeting of locus transcription to mRNA. Stimulation of StAR loci by Br-cAMP produced immediately and delayed (after 60 minutes) responses. The immediate response was associated with increases of p-RNA at activated loci followed by increases in sp-RNA but with no increase in mRNA. The presence of p-RNA indicates slow splicing which we described as an uncoupled transcription. After 60 minutes, while the locus p-RNA and sp-RNA remained unchanged, cytoplasmic mRNA suddenly increased. This absence of change at the loci indicated that a proportion of loci had switched to the generation of mRNA without intermediate pRNA (splicing is coupled to transcription). Br-cAMP stimulation 4 hours after CRISPR transfection resulted in the Cas9 elimination of the uncoupled activation but not the coupled mRNA formation. The increase in StAR protein after 60 minutes (Figure 2D) suggests that mRNA was forming but remained undetected. Protein/ribosomal complexes may limit access to FISH probes. gRNA Cas9 may gain access to the more open rapidly transcribed loci.
